# Design and implementation of the fractional-order controllers for a real-time nonlinear process using the AGTM optimization technique

**DOI:** 10.1038/s41598-024-82258-1

**Published:** 2024-12-30

**Authors:** Sabavath Jayaram, Nithya Venkatesan

**Affiliations:** School of Electrical Engineering, Vellore Institute of Technology, Chennai, 600127 India

**Keywords:** Nonlinear system, First order plus time delay, AGTM optimization, Fractional-order controllers, Performance indices, Time domain specifications, Chemical engineering, Electrical and electronic engineering

## Abstract

Spherical tanks have been predominantly used in process industries due to their large storage capability. The fundamental challenges in process industries require a very efficient controller to control the various process parameters owing to their nonlinear behavior. The current research work in this paper aims to propose the Approximate Generalized Time Moments (AGTM) optimization technique for designing Fractional-Order PI (FOPI) and Fractional-Order PID (FOPID) controllers for the nonlinear Single Spherical Tank Liquid Level System (SSTLLS). This system features a large dead time, and its real-time modeling generally represents a Single Input Single Output (SISO) model. However, in practice, the derived SISO model is often a First Order Plus Dead Time (FOPDT) model, necessitating an effective controller to maintain the tank’s steady-state level. In this research, the proposed AGTM method, based on the conventional Proportional Integral (PI) and Proportional Integral Derivative (PID) controllers, is compared with the FOPI and FOPID controllers for the nonlinear SSTLLS. The performance of these controllers is contrasted using metrics such as Integral Squared Error (ISE) and Integral Absolute Error (IAE), as well as time-domain characteristics containing Rise time, Peak time, Settling time, Peak overshoot, and Steady-state error. The implementation of the aforementioned controllers is done in simulation and real-time employing the MATLAB software environment and the Data Acquisition (DAQ) device National Instrument NI-DAQmx 6211. The simulation and experimental results demonstrate the exceptional performance of the designed Fractional-Order controllers based on the proposed method which offers an increased degree of freedom despite the more complex design process.

## Introduction

Process industries face challenges in controlling dynamic parameters, such as unpredictable changes, limited manipulated variables, and delayed responses. Traditional control techniques are crucial for managing systems with large dead times and nonlinearity. Spherical tanks, known for their large storage capacity, present unique challenges due to their nonlinear behavior, requiring precise control to prevent disruptions. Advanced sensors, automation, and tailored control strategies are vital for maintaining stability and efficiency. The PID controller is widely used in process control for its simplicity and self-tuning capability, although tuning it for specific needs can be challenging. This has led to the development of advanced strategies like Model Predictive Control (MPC), Fractional-Order controllers, fuzzy logic, and machine learning. The subsequent literature outlines various control methodologies tailored to different spherical tank arrangements. Sundaravadivu et al.^1^ developed the FOPID controller to regulate liquid levels in a spherical tank. Building on this, Nithya et al.^2^ employed a cost-effective ADAM data acquisition module for real-time data collection and implemented a soft computing-based PI controller for the spherical tank process. Similarly, using the same data acquisition card, Nithya et al.^3^ conducted a real-time comparison between a fuzzy logic controller and a PI regulator for the spherical tank. Baruah et al.^4^ introduced the FOPI controller for a Coupled Tank System (CTS), incorporating online relay auto-tuning through the state space method. Vázquez et al.^5^ developed the FOPID controller for a two-tank system, using the Genetic Algorithm (GA) for optimization. Additionally, Pannem et al.^6^ designed and validated PI and FOPI controllers for real-time SSTLLS at various operating points. Finally, Chakravarthi and Venkatesan^7^ developed PI controllers for SSTLLS in real-time using a LabVIEW environment. Fractional-order controllers provide greater flexibility with the integral (λ) and derivative (μ) terms, enabling more accurate real-time control and reducing sensitivity to process changes. They effectively manage both integer-order and fractional-order dynamics, enhancing accuracy in nonlinear systems.

Recent advancements in the FOPID controller design and application have revolutionized a broad spectrum of engineering systems. Researchers have proposed innovative enhancements in FOPID controllers across various domains, from aerospace to robotics and renewable energy systems. Yang et al.^8^ enhanced a FOPID controller for Electromechanical Actuation systems (EMAs) in aircraft using Particle Swarm Optimization (PSO), integrating current feedforward to resolve force conflicts in fly-by-wire systems. Chen and Luo^9^ took a different approach, designing a FOPID controller for Permanent Magnet Synchronous Motor (PMSM) speed servo systems with a Bode’s Ideal Cutoff (BICO) filter, optimizing performance and stability. In another PMSM application, Zheng et al.^10^ applied a synthesis approach to create a FOPID controller for speed control, focusing on precise performance metrics. Birs et al.^11^ tackled auto-tuning techniques for FOPID controllers in Vertical Take-Off and Landing (VTOL) platforms, using methodologies such as the Sine-Test, FO-KC, and FO-ZN within LabVIEW, to enhance control system efficiency. Robotics and automation also benefit from these advancements. Huang and Chuang^12^ designed a FOPID controller for real-time Machine Learning Control (MLC) in robotic manipulators, using the Artificial Bee Colony (ABC) algorithm for optimization. In a related field, Vanchinathan and Selvaganesan^13^ employed the ABC algorithm for FOPID control in Brushless DC (BLDC) motors, improving dynamic response and stability. The FOPID controller’s utility extends to unstable and nonlinear systems as well. Mondal and Dey^14^ successfully implemented it for a cart-inverted pendulum system, using the root locus method for stabilization. In power systems, Mishra et al.^15^ improved power quality in Hybrid Shunt Active Power Filters (HSAPF) by using a FOPID controller combined with the hybrid PSO-Grey Wolf Optimization (PSO-GWO) technique. Maddahi et al.^16^ introduced a model-free FOPID controller for hydraulic actuators, employing Iterative Feedback Tuning (IFT) to achieve robust control without requiring system modeling. Similarly, in control systems, Acharya et al.^17^ utilized the Hermite-Biehler theorem and the Multiagent-based Symbiotic Organisms Search (MASOS) algorithm to stabilize a second-order unstable Magnetic Levitation Plant (MLP) with time delays, advancing control in complex systems. Swain et al.^18^ also worked with MLPs, designing the FOPID controller using dominant pole placement and enhancing the objective function through nonlinear interior point optimization. Expanding on this, Laifa et al.^19^ developed a FOPID controller for both SISO and MIMO systems, based on the Smith predictor structure, using a direct synthesis method. In a similar vein, Boudjehem et al.^20^ created a FOPID controller for a process with dead-time, incorporating a GA within the Fractional-order Smith predictor structure framework. For Automatic Voltage Regulators (AVR), Tumari et al.^21^ introduced a FOPID controller optimized through the Marine Predators Algorithm (MPA), while G. Babu and Chiranjeevi^22^ employed the Genetic Algorithm (GA) and Ant Colony Optimization (ACO) to fine-tune the controller for dynamic performance improvement in AVRs. In HVAC systems, Li et al.^23^ developed the FOPID controller with a Modified Ant Colony Optimization Algorithm (MACOA) for precise temperature regulation in air-conditioned rooms using Fan Coil Units (FCUs). Likewise, Fu and Lu^24^ adapted a FOPID controller for Hydraulic Turbine Governing Systems (HTGS), employing Harris Hawks Optimization (HHO) to enhance control dynamics. Jain and Hote^25^ focused on a DC servo system, refining the traditional Ziegler-Nichols (ZN) method with Big Bang Big Crunch (BBBC) optimization to achieve better control precision. Ashjaee and Tavazoei^26^ tackled thermal furnace systems using optimal tuning strategies to optimize the FOPID controller, ensuring energy efficiency and system stability. In the medical robotics field, Li et al.^27^ applied a FOPID controller to a Cable-Driven Waist Rehabilitation Robotic System (CDWRRS), using the enhanced Oustaloup numerical approximation method to improve rehabilitation accuracy and patient outcomes.

This research elaborates on the AGTM framework, elucidating its principles and demonstrating its application in optimizing both Fractional-Order and Integer-Order controllers for SSTLLS. It highlights the adaptability, stability, and effectiveness of AGTM in addressing the complexities inherent in these systems. The significance of Time Moments (TM) and Markov Parameters (MP) during controller development is discussed in^28^, focusing on aligning the transient and steady-state responses of both the reference system and the closed-loop structure. The optimal amalgamation of TM and MP for critically damped and underdamped structures is investigated in^29^. Jayaram and Venkatesan^30^ designed FOPID controllers for liquid-level regulation in SSTLLS using the AGTM optimization technique. However, despite the simplicity of this design approach, automating the entire process remains challenging due to the need for expansion and inversion. Pal^31^ introduced a novel method for reducing model order in the frequency domain by introducing two variables, AGTM and Approximate Generalized Markov Parameters (AGMP). This technique creates a steady lower-order structure for higher-order systems through frequency combining, offering a range of reduced-order models from various expansion points for users to select a stable option. In conclusion, unlike the other quoted papers, the proposed AGTM method imposes no restrictions on the model’s order or the structure of the controller transfer function. A precise reference model can be fully utilized and identified using the given time domain parameters to avoid stabilization issues in the spherical tank. The AGTM method is computationally simple, easy to implement, ensures closed-loop system stability, meets performance criteria, and computes quickly.

The present work is organized as follows: The experimental process and model description for the SSTLLS are presented in the Section “Experimental process description”. The design of the controllers is discussed in the Section “Design of the controllers”. A discussion of the proposed method for controller design is provided in Section “The proposed method for controllers design”. The outcomes from implementing the AGTM optimization method for controller design in SSTLLS, along with an analysis of the simulation and experimental results, are presented in Section “Results and discussion”. The key findings and contributions of the research are highlighted in the final Section “Conclusion”, which emphasizes the significance of the AGTM optimization method in addressing controller design issues for SSTLLS and offers insights into potential future research directions in this field.

## Experimental process description

**Fig. 1:**
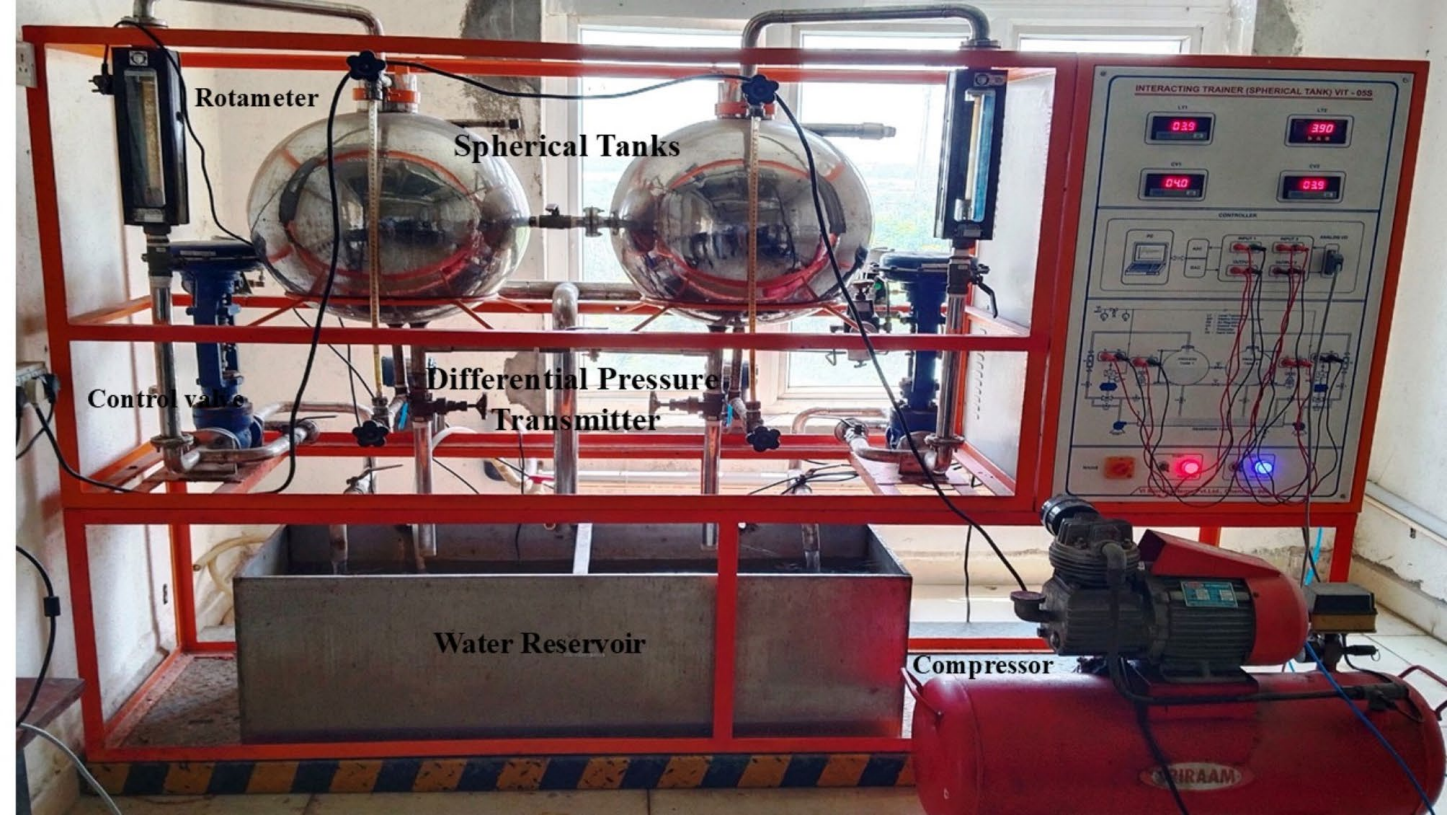
Real-time experimental arrangement of the process.

In the laboratory setup, two interacting spherical tanks are connected by a manually operated valve. Water is pumped into and out of both tanks from a reservoir, with flow controlled by pneumatic valves adjustable via air pressure from a compressor. A rotameter is used to manually set the flow rate. Tank levels are measured using a differential pressure transmitter with a 4–20 mA output. The NI-DAQmx 6211 data acquisition card, with a 10-V range, 16 analog inputs, and 2 analog outputs, connects the transmitter to the computer. The card has a sampling rate of 250 kS/s and a resolution of 16 bits. The process’s real-time experimental setup is depicted in Fig. 1.

**Fig. 2:**
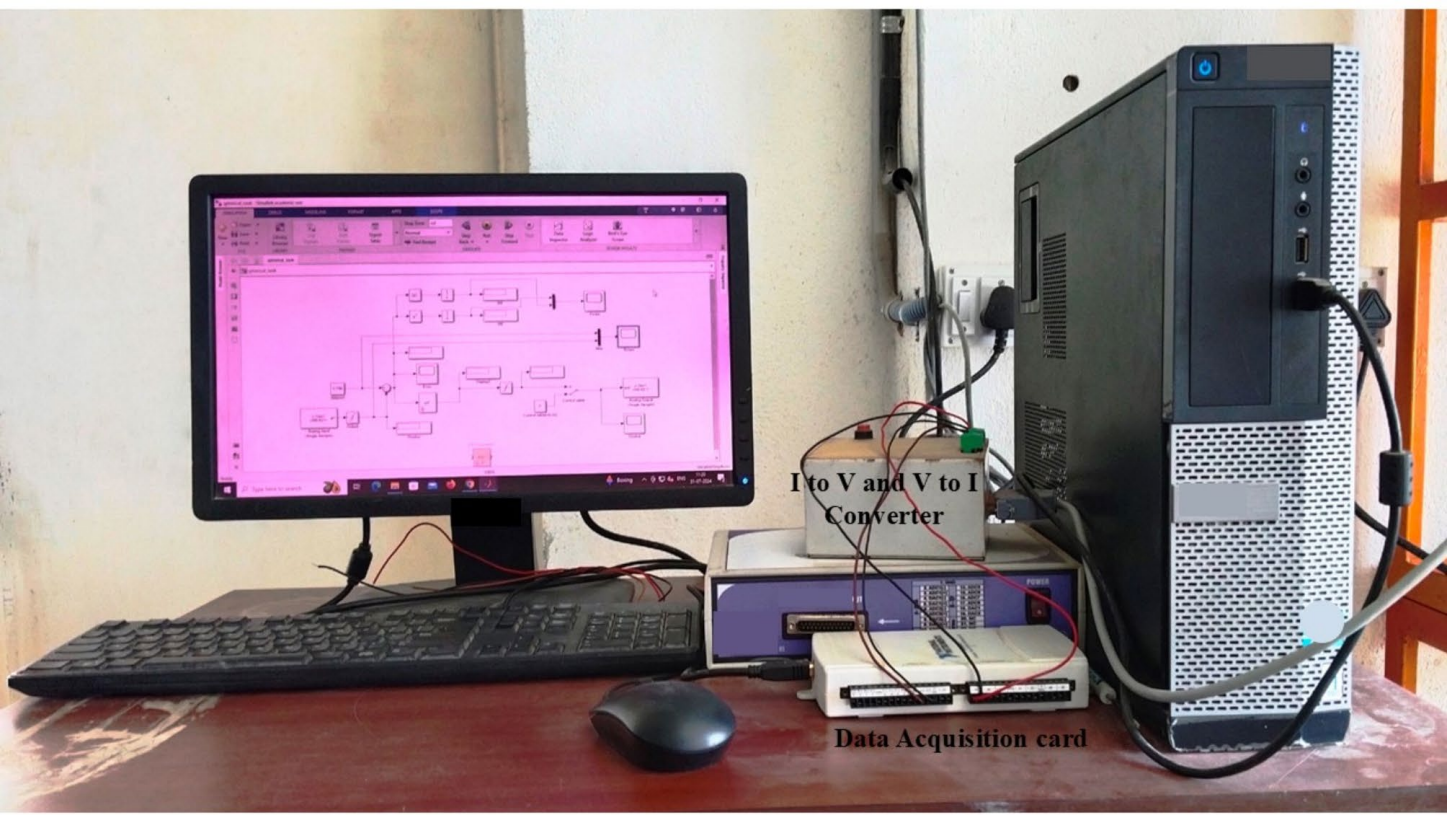
The connected NI-DAQmx 6211 DAQ module device.

**Fig. 3:**
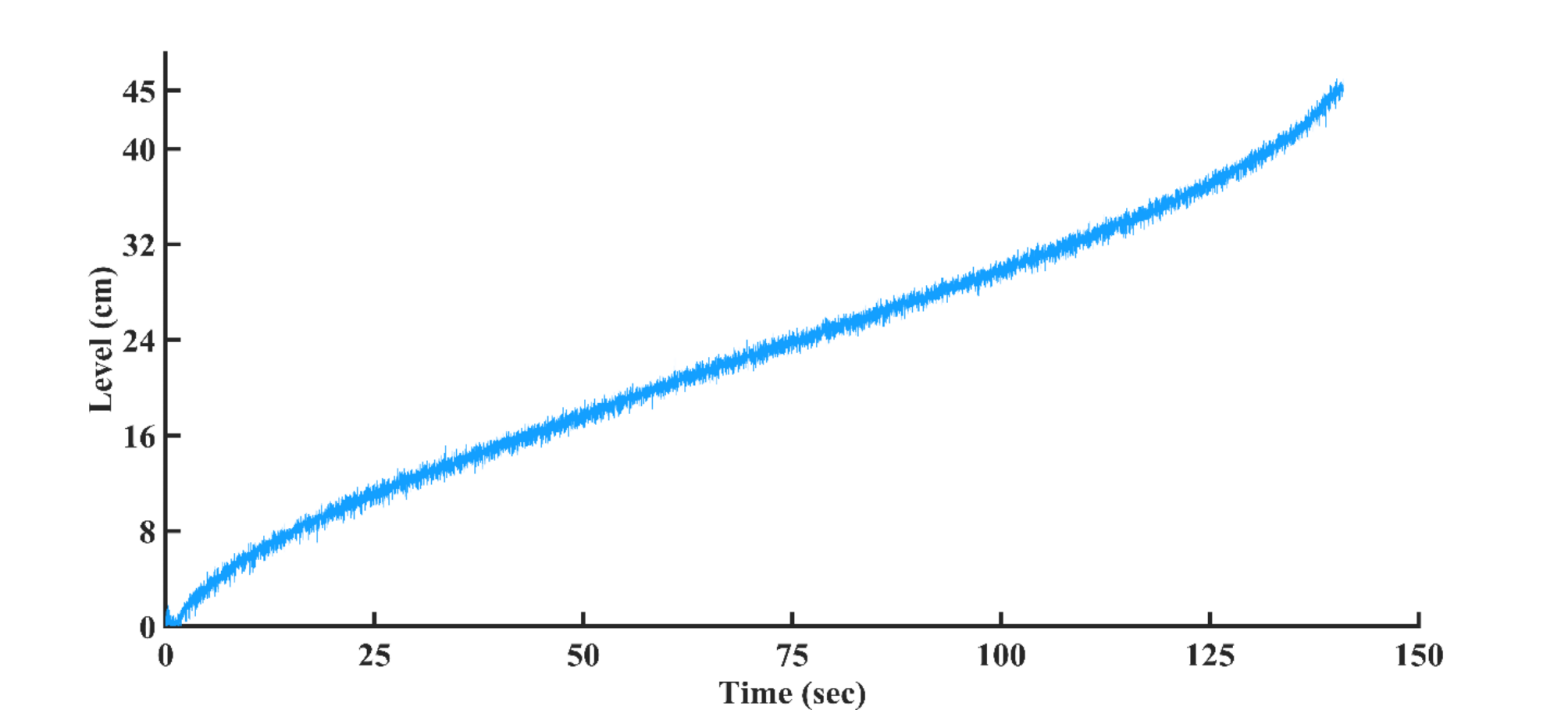
S-shaped open loop input-output performance graph.

When the pneumatic control valve closes, the process begins by adjusting the water flow into the tank using air. The NI-DAQmx 6211 data acquisition card collects data from the differential pressure transmitter, which measures water volume using a 4–20 mA current range. The computer calculates the control strategy and sends the signal to the I/P converter, which then adjusts the pneumatic valve to regulate water flow into the tank. The connected NI DAQmx 6211 data acquisition cards are depicted in Fig. 2. Technical details of the connected spherical tanks are presented in Table 1. This paper derives the plant model experimentally using a step input. By analyzing the data from this input, we created an accurate model of the system dynamics. The step response curve, shown in Fig. 3, may have an S-shape if the system lacks integrators or complex conjugate poles. The time constant and delay time are two constants used to characterize the S-shaped curve. Following the derivation of the model, we proceeded to develop and implement PI, PID, FOPI, and FOPID controllers. These controllers depend on the AGTM optimization approach to regulate the SSTLLS level parameters in real-time via the data acquisition device.

**Table 1:** Technical details of the experimental configuration.

Hardware components	Details
Double spherical tanks	Material: Stainless steel
	Diameter: 45 Cm
Differential pressure transmitter	Type: Capacitance
	Range: (2.5 to 250) Mbar
	Output: (4–20) mA
	Make: ABB
Control regulator	Size:1/4", Pneumatic
	Actuated type: Air to close
	Input: (3–15) PSI
	0.2–1 kg/cm2
Air regulator	Size:1/4" BSP
	Span: (0–2.2) BAR
Pump	Centrifugal 0.5 HP
Input converter	Input: (4–20) mA
	Output (3–15) PSI
Pressure gauge	Span: (0–30) PSI
	Span: (0–100) PSI

### Mathematical modeling of SSTLLS

The spherical tank process is notably nonlinear, primarily due to the tank’s diameter. Experimental findings show that diameter variations lead to system nonlinearity, explained by a first-order differential equation. The mathematical model of the liquid-level system is derived using the principle of mass balance.

1 $$\frac{{\text{Accumulation of total mass}}}{{{\text{time}}}} = \frac{{\text{input of total mass}}}{{{\text{time}}}} - \frac{{\text{output of total mass}}}{{{\text{time}}}}$$

The single spherical tank model can be expressed as

2 $$\frac{\text{d}V}{\text{d}t}={Q}_{1}-{Q}_{2}$$

where $V=\frac{4}{ 3}\pi {h}^{3}$ is the volume of the tank and it is directly proportional to the cube of the radius of the tank, *h* is the height of the tank in cm, ${Q}_{1}$ is the inlet flow rate and ${Q}_{2}$ is the outlet flow rate. When steady-state values are utilized and Eqs. (2) along with the tank’s volume are solved, the nonlinear spherical tank can be simplified into the subsequent linearized model,

3 $$\frac{H(s)}{{Q}_{1}(s)}=\frac{{R}_{t}}{\tau s+1}$$

where ${R}_{t}=\frac{{2h}_{s}}{{q}_{2}(s)}$, $\tau =4\pi {R}_{t}{h}_{s}$ is the time constant and ${h}_{s}$ is the height of the tank at the steady state. Real-time structure identification of the SSTLLS is achieved through the utilization of a black-box modeling approach. The tank undergoes a filling process from 0 to 45 cm with consistent water inflow and outflow rates. The NI-DAQmx 6211 acquires individual samples through the USB port from the differential pressure transmitter, operating within the 4 to 20 mA range. Later, this data is transmitted to the personal computer. The collected information is transformed into centimeter-based measurements. By employing the open-loop technique, the system’s reaction to changes in the input is documented. Sundaresan and Krishnaswamy^32^ determined ${\tau }_{p}$ and $\theta$ by matching the process’s open-loop response to the model at two specific time points. Using a step response, the times ${t}_{1}$ and ${t}_{2}$, corresponding to 35.3% and 85.3% response durations, are used to calculate the time constant and delay,

4 $${\tau }_{p}=0.67({t}_{2}-{t}_{1})$$

5 $$\theta =1.3{t}_{1}-0.29{t}_{2}$$

with constant inlet and outlet flow rates, the process stabilizes at a steady state. Following this, a step increment is introduced by altering the flow rate, and multiple values are recorded until the system stabilizes once more, as depicted in Fig. 3. From this investigation, an open loop response of the structure is obtained and thereby the parameters of ${t}_{1}$ and ${t}_{2}$ are noted as 45.1760 s and 130.5360 s respectively. The empirical data are estimated to follow a FOPDT model, represented by Eq. (6) as follows:

6 $$G\left(s\right)=\frac{1.388}{57.19s+1}{e}^{-20.9s}$$

## Design of the controllers

The system’s transfer function model enables the design of a controller to maintain the ideal setpoint, achievable through careful selection and tuning of parameters. Tuning methods express these parameters as equations or algorithms, ensuring a reliable control system. Significant improvements in stabilizing techniques have also been made.

### Proportional integral derivative (PID) controller

PID controllers have long been central to control systems, widely used in industrial automation due to their simplicity, reliability, and ease of tuning. They account for approximately 95% of control mechanisms, proving highly adaptable across various applications. Their flexible design allows for optimal performance in diverse processes, making them a go-to solution in industrial settings.

**Fig. 4:**
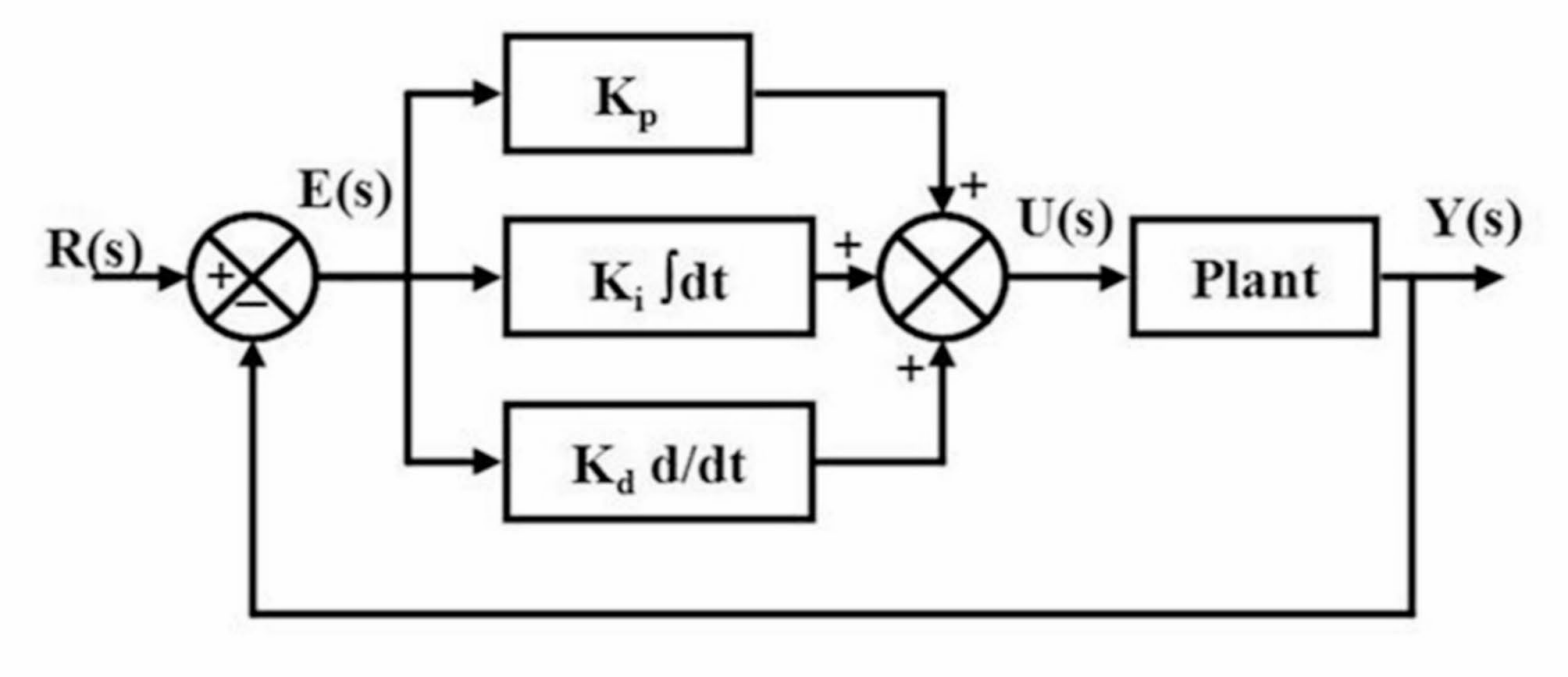
The general structure of the PID controller with a closed-loop system.

Three adjustable control parameters here are proportional control ${K}_{p}$, integral control ${K}_{i}$, and derivative control ${K}_{d}$ gains. Figure 4 shows a closed-loop system with a PID controller. The mathematical expression defining the PID controller is presented as follows,

7 $${G}_{c}(s)={K}_{p}+\frac{{K}_{i}}{s}{+K}_{d}s$$

### Fractional-order PID (FOPID) controller

**Fig. 5:**
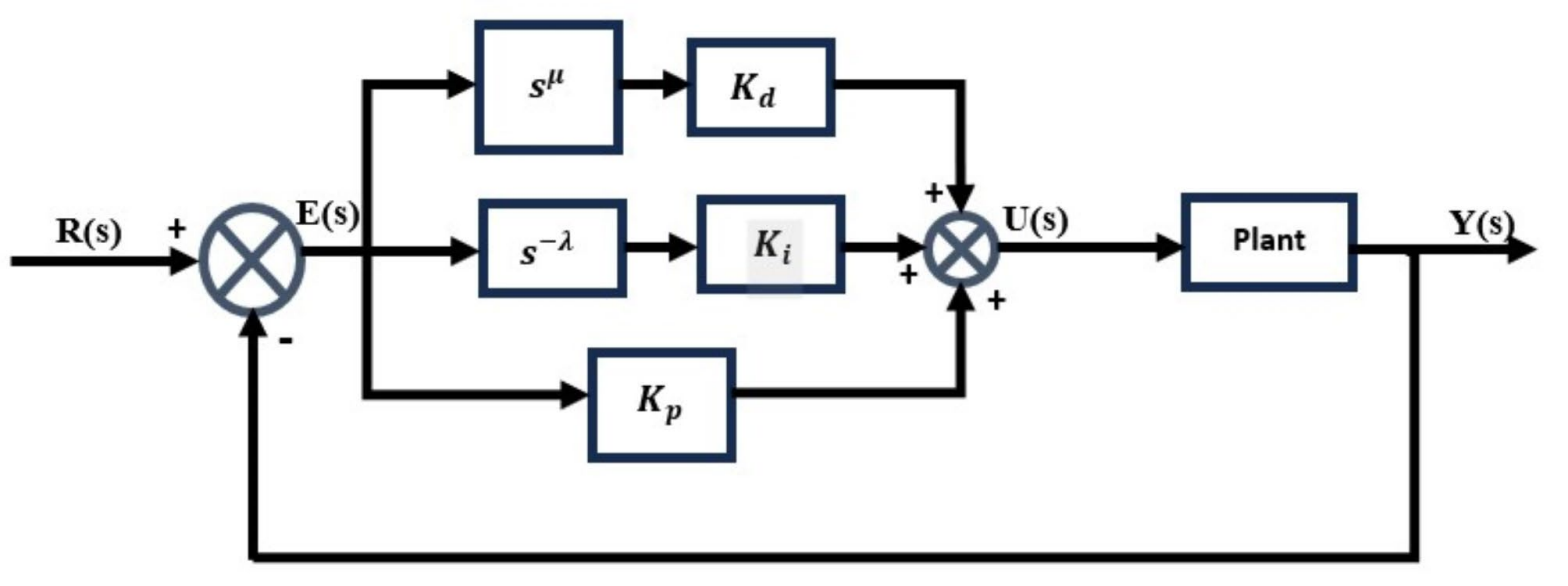
The general configuration of the closed-loop structure employing the FOPID controller.

Fractional calculus has gained attention for its unique properties and applications, especially in control systems. Fractional-Order controllers, like the FOPID, offer enhanced performance over traditional PID controllers by tuning five parameters instead of three, improving robustness and control. This note highlights the advantages of FOPID controllers, emphasizing their ability to deliver superior control precision, stability, and disturbance rejection. Figure 5 shows a closed-loop structure with the FOPID controller, with its typical transfer function presented as^33^,

8 $${{G}_{c}\left(s\right)=\frac{U(s)}{E(s)}=K}_{p}+\frac{{K}_{i}}{{S}^{\lambda }}+{K}_{d}{s}^{\mu } ;\hspace{1em}\lambda <2,\upmu <2$$

In this context, ${G}_{c}(s)$ symbolizes the transfer function of the produced controller, while $U\left(s\right)$ and $E\left(s\right)$ stand for the control input and output error signals, respectively. ${K}_{p}$, ${K}_{i}$, and ${K}_{d}$ denote the proportional, integral, and derivative coefficients, while *λ* and *μ* indicate the fractional aspects of the integral and derivative components, respectively. Fractional-Order systems can only be simulated by approximating them to integer-order systems. Various approximation methods exist, but Charef and Oustaloup’s^34,35^ techniques are the most well-known and effective in the frequency domain.

In this paper, the ${s}^{\mu }$ term of the Fractional-Order differentiator and Fractional-Order integrator are approximated using Oustaloup’s method as follows

9 $${s}^{\mu\:}\:\cong\:{s}_{\left[{w}_{b},{w}_{h}\right]}^{\mu\:}\cong\:K\prod\:_{i=1}^{N}\frac{s+{w}_{i}^{{\prime\:}}}{s+{w}_{i}}$$

where $N$ represents the order of approximation and ${w}_{b}$, ${w}_{h}$ denote the lower and upper bounds of the frequency approximation interval. The gain, poles, and zeros can be calculated as follows:

10 $$K=Gain={w}_{h}^{\mu }$$

11 $${w}_{i}{\prime}=Zeros={w}_{b}{\left(\frac{{w}_{h}}{{w}_{b}}\right)}^{\frac{i+N+\frac{1}{2}(1-\mu )}{2N+1}}$$

12 $${w}_{i}=Poles={w}_{b}{\left(\frac{{w}_{h}}{{w}_{b}}\right)}^{\frac{i+N+\frac{1}{2}(1+\mu )}{2N+1}}$$

## The proposed method for controllers design

### Preliminaries

 Preliminaries Advancements in control design for nonlinear systems have been propelled by model matching techniques, particularly Exact Model Matching (EMM) and Approximate Model Matching (AMM). EMM requires the model’s transfer function to have at least as many poles as the plant’s for a realizable controller. AMM resolves this issue by enabling compensator design through optimization techniques, without restrictions on order. This research employs AMM to achieve the specifications of a general reference transfer function. The Padé approximation, introduced by Padé^36^ in 1890, is commonly used for model reduction and compares the time moments and Markov parameters of the plant’s lower-order model. However, it does not guarantee stability for the reduced model or the closed-loop system. Pal^31^ offers a partial solution by introducing a more comprehensive set of parameters than time moments and Markov parameters. To cover the entire frequency range of interest, one may match the expansion coefficients around ${s=\delta }_{i}$ rather than $s=0$ and $s=\infty$, where ${\delta }_{i}$ are selected to be large or small, real or complex integers. Consequently, Approximate Generalized Markov Parameters (AGMP), also known as AGTM, was introduced in^37–41^ as a controller or model design technique across various applications. The GA optimization approach is employed to identify optimal expansion points for the AGTM/AGMP method. GA is a stochastic method inspired by natural selection and was introduced by John Holland et al.^42^ in 1975. Key genetic operations in GA include crossover, recombination, mutation, and selection. The FOPID controller design in this study is computationally simple, uses only output feedback, and results in a low-order, practical controller without requiring plant order reduction. Thus, leveraging these benefits, the AGTM method addresses the FOPID controller design for nonlinear systems.

### Approximate generalized time moments (AGTM) technique

**Fig. 6:**
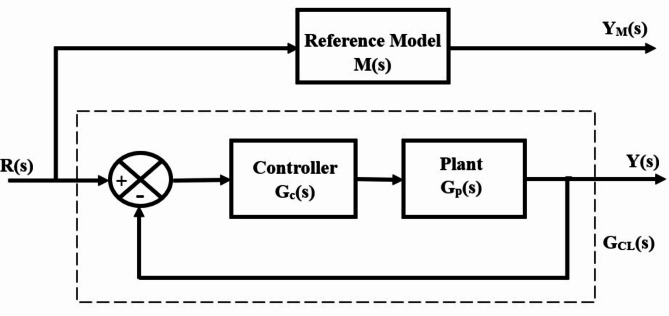
Block schematic of the AGTM methodology.

The objective of the AGTM method is to identify the features of the unknown controller ${G}_{c}(s)$ in a manner that aligns with the response of the reference model $M\left(s\right)$ and the complete closed-loop system ${G}_{CL}(s)$. This reference model is selected to reflect the desired time-domain characteristics of the closed-loop structure. Choosing the controller’s parameters is essential for achieving optimal similarity between the closed-loop structure and the reference model. Consider a general higher-order time delay structure defined by the following transfer function,

13 $${G}_{p}(s)=\frac{{b}_{m-1}{s}^{m-1}+{b}_{m-2}{s}^{m-2}+\text{.....}+{b}_{0}}{{s}^{m}+{a}_{m-1}{s}^{m-1}+{a}_{m-2}{s}^{m-2}+\text{.....}+{a}_{0}}{e}^{-{\tau }_{d}s}$$

in the block schematic illustrated in Fig. 6, ${G}_{p}\left(s\right), M\left(s\right)$, and ${G}_{CL}(s)$ represent the plant, reference model, and the complete closed-loop system, correspondingly. The reference model transfer function was produced in the manner previously mentioned to satisfy the closed-loop system’s required time-domain characteristics. Subsequently, the reference model transfer function is expressed as follows,

14 $$M\left( s \right)\; = \;\frac{{\omega _{n}^{2} }}{{s^{2} + 2\xi \omega _{n} s + \omega _{n}^{2} }}e^{{ - T_{d} s}}$$

Now, the controller parameters need meticulous selection to achieve the closest possible resemblance between the closed-loop structure and the reference model. This can be accomplished by amalgamating multiple AGTMs of $M\left(s\right)$ and ${G}_{CL}(s)$ at different extension points, creating a series of linear equations where the unknown aspects represent the controller parameters. The resulting closed-loop process transfer function turns out to be

15 $${G}_{CL}(s)=\frac{Y(s)}{R(s)}=\frac{{G}_{c}(s){G}_{p}(s)}{1+{G}_{c}(s){G}_{p}(s)}$$

In the AGTM-based controller design, the objective is to ensure that the closed-loop structure transfer function aligns with the reference model transfer function, depicted as follows,

16 $${\left.M(s)\right|}_{s={\delta }_{i}}\cong {\left.{G}_{CL}(s)\right|}_{s={\delta }_{i}}$$

The time moments of the closed-loop system ${G}_{CL}(s)$ are coordinated with those of the reference model *M(s)* at numerous frequency points ${\delta }_{i}$, where *i* = *1,2,3….* ${n}_{e}$ and ${n}_{e}$ is the number of undisclosed controller variables that are required to be identified as

17 $$M(s)=\frac{{G}_{c}\left(s\right){G}_{p}\left(s\right)}{1+{G}_{c}\left(s\right){G}_{p}\left(s\right)}{|}_{s={\delta }_{i}}$$

Using the above equation, we get

18 $${G}_{c}(s)=\frac{M(s)}{{G}_{p}(s)-M(s)\text{*}{\text{G}}_{p}(s)}{|}_{s={\delta }_{i}}$$

to determine the unknown variables of the designed controllers. They can be directly obtained by solving the set of nonlinear equations in the *s-*domain using the AGTM method over the set of expansion points. The total number of undefined variables of the controllers can be found by resolving the preceding nonlinear equation, which involves choosing the expansion points, or a set of values for ${s=\delta }_{i}$. The objective is to optimize the performance index Integral Squared Error (ISE) value to achieve the highest degree of similarity among the responses of the reference model and the closed-loop structure controlled by the designed controller. Typically, the optimization challenge is formulated as.

find ${\delta }_{i}$, *i* = *1,2,3. .* .${n}_{e}$ so as to reduce:

19 $$\text{ISE}={\int }_{0}^{t}({y}_{m}(t)-{y}_{cl}(t){)}^{2}\text{d}t$$

adhering to the limitations: the actual components of the poles within the closed-loop structure must lie on the left-hand side of the *s*-plane.

20 $$\{ {\text{real }}[{\text{pole}}] G_{{CL}} \left( s \right) \} \, < \,0$$

**Fig. 7:**
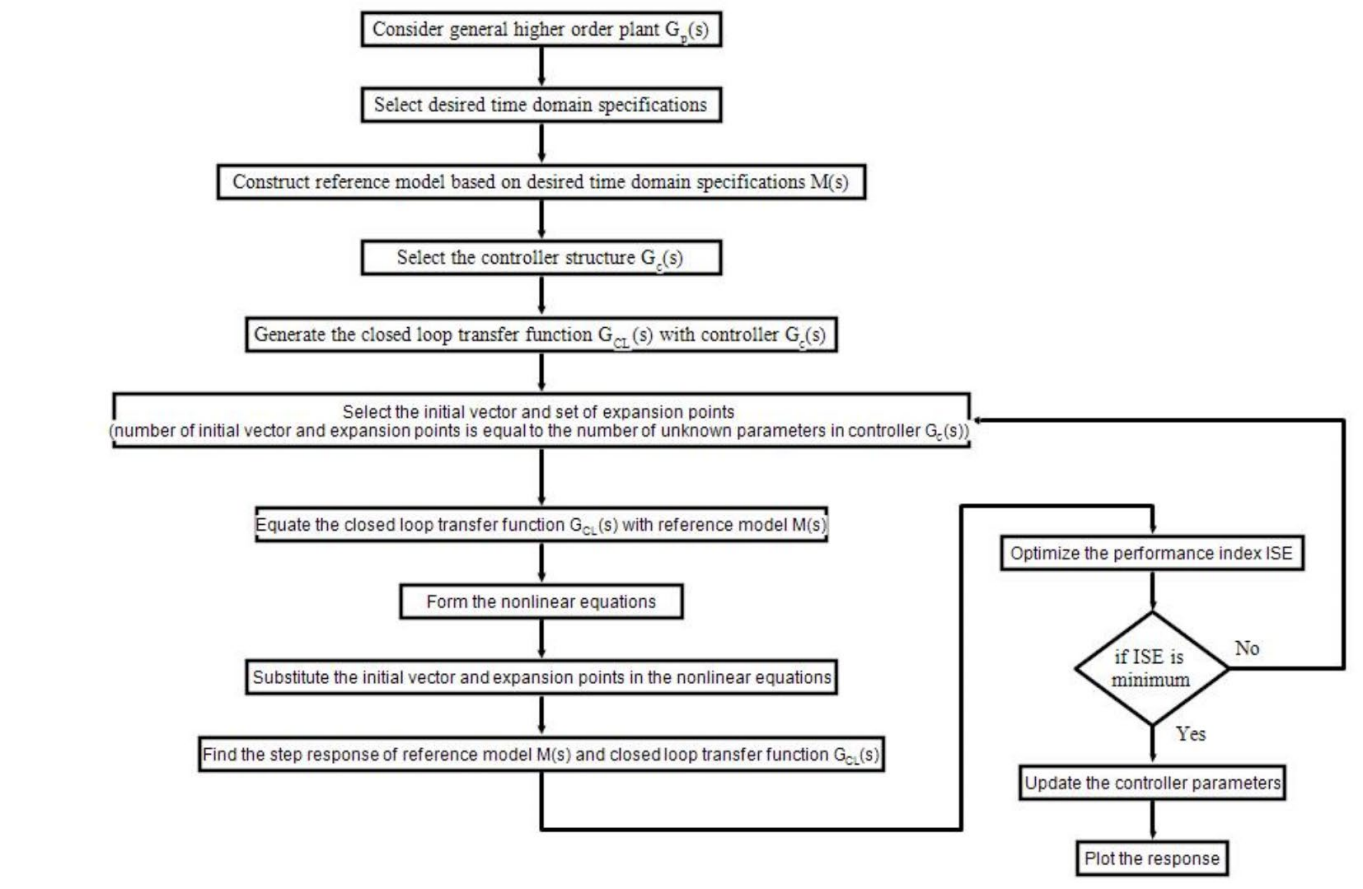
Flow chart of the AGTM optimization algorithm.

In this context, ${y}_{cl}(t)$ denotes the output of the closed-loop structure governed by the suggested controller ${G}_{CL}(s)$, while ${y}_{m}(t)$ represents the outcome of the reference model $M\left(s\right)$. Controller variables are selected by minimizing the ISE value through adjustments to the initial vector, expansion points, or both. The ISE criterion aims to minimize the sum of squared errors over time, penalizing larger deviations more than smaller ones, making it effective in systems where large errors are undesirable. By squaring the errors, ISE significantly impacts the total value, ensuring effective control performance in tuning the Fractional-Order controllers for the spherical tank. This helps maintain the liquid level close to the desired setpoint with minimal overshoot and settling time, which is crucial for system performance and safety. Prioritizing larger error reduction allows the controller to quickly correct significant deviations, enhancing stability and reliability. Smoother control actions, facilitated by ISE, reduce wear on equipment and improve system stability, especially in response to rapid control changes. The straightforward ISE formula is easy to implement, assisting the optimization process in tuning Fractional-Order controllers, where the Fractional Orders add complexity to the parameter space. By leveraging additional tuning parameters with ISE, robust and high-performance liquid-level control in spherical tanks is achievable. In the AGTM approach, the expansion points ${\delta }_{i}$ are determined using a GA to minimize ISE performance indices, ensuring a robust match between the reference model and the designed responses. The GA adheres to the stability constraint outlined in Eq. (20), promoting resilience in the closed-loop system from the chosen expansion points. Figure 7 displays a flowchart of the AGTM technique for the controller, illustrating how the algorithm optimizes it.

### Controller design process using AGTM

 AGTM is a model-matching optimization technique, and grasping its principles, algorithms, and parameters is essential for effective controller design, necessitating familiarity with mathematical optimization methods and AGTM’s specific approach.

Selection of the Reference Model: Selecting a reference model is essential in controller design, as it dictates the interaction within the controlled dynamics. The desired closed-loop reference model is selected based on design criteria and process dynamics to ensure it meets system specifications. The model’s dynamics must be realizable with an achievable controller. In the AGTM optimization approach, a general model transfer function is chosen to satisfy predefined performance criteria, with no limits on order or time delay. The goal is to determine the optimal interaction factor that meets specific time domain output requirements.

Selection of Expansion Points: Expansion points can be positive or negative real numbers or complex points from any quadrant of the *s*-plane. The minimum number of expansion points usually matches the number of unknown controller parameters. When choosing complex points, it’s important to avoid conjugate pairs. These points are vital for maintaining stability and optimizing closed-loop system performance, ensuring the design meets specifications. Currently, there is no established theory for selecting expansion points to keep the closed-loop system’s poles in the left half of the *s*-plane. This study approaches the selection of optimal expansion points for a stable response as a constrained optimization problem, addressed using a GA, as outlined in the previous section.

Fractional-Order Dynamics: Fractional-Order controllers utilize fractional calculus, enabling non-integer orders of differentiation and integration. The gain is restricted between 0 and 10, while the fractional operator ranges from 0 to 2. These limits, set by the algorithm, aim to balance precision and adaptability.

Objective Function: The objective function for selecting the optimal decision variables is defined as the integral of squared deviations in the step response of the designed closed-loop system from that of the reference model across a chosen simulation period. The AGTM technique endeavors to ascertain the unknown controller ${G}_{c}(s)$ variables such that the response of the reference model $M\left(s\right)$ aligns with that of the overall closed-loop transfer function $M\left(s\right)$.

Boundary constraints: In a closed-loop system, the poles’ real parts must be positioned in the left half of the *s*-plane. The dynamics of spherical tanks can be challenging to model and control due to nonlinearities, time delays, and uncertainties. System parameter uncertainties and disturbances can degrade control performance, making it difficult to meet performance requirements like settling time, overshoot, and steady-state error with conventional methods. The AGTM optimization technique is essential for addressing these challenges, offering a systematic approach to designing controllers for nonlinear systems using generalized time moments and Markov parameters. AGTM effectively manages complexities, time delays, and uncertainties, leading to improved controller performance. Incorporating generalized time moments allows for precise tuning of the controller’s response to meet specifications for overshoot, settling time, rise time, and stability margins—vital for nonlinear system performance. The motivation for utilizing AGTM optimization techniques for controller design in SSTLLS stems from the necessity for robust and effective control solutions capable of addressing the inherent complexities and challenges of such a system.

## Result and discussions

The purpose of this experimental effort was to assess the performance and disturbance rejection capabilities of different controllers utilized in SSTLLS as shown in Fig. 1. The controllers employing the proposed AGTM optimization method were tested in simulation and real-time across various setpoints to assess their efficacy in managing liquid levels within the system. The controllers’ performance, including tracking accuracy, response time, overshoot, and settling time, was measured and compared across different setpoints and disturbances. As per the details outlined in the reference^43^, the values assigned to the parameters, such as Delay time, Rise time, settling time, and Damping ratio, are specified as 20.9 s, 0.229 s, 83.5142 s, and 0.75, correspondingly. The reference model’s transfer function is derived based on these specifications, presented as follows,

21 $$M(s)=\frac{100}{{s}^{2}+15s+100}{e}^{-20.9s}$$

The GA determines the optimal expansion points using a population size of 100, a crossover fraction of 0.75, an initial population range of 0.1 to 1, and a total of 100 generations. Performance metrics, defined by Eq. (20), measure the area between the intended and designed responses. The goal is to minimize the performance index ISE to align the designed response closely with the specified response. The derived parameters of the various employed controllers are shown in Table 2.

**Table 2:** Designed controller variables.

Type of controller	Set of expansion points	Initial vector	Controller parameters				
			$\:{K}_{p}$	$\:{K}_{i}$	$\:{K}_{d}$	$\:\lambda\:$	$\:\mu\:$
PI	[0.0281 0.0246]	[0.01 0.01]	1.0923	0.0337	-	-	-
PID	[0.00026 4.855 0.0016]	[0.5 0.5 0.5]	2.3143	0.0342	21.1120	-	-
FOPI	[0.0274 0.557 0.297]	[0.01 0.01 0.01]	2.6541	1.6935	-	0.8085	-
FOPID	[1.04994 1.3655 3.08409 1.04131 1.03289]	[0.5 0.5 0.5 0.5 0.5]	3.2945	0.6450	3.8051	0.7624	0.1023

After determining the five parameters of the FOPID controller, its actual realization structure is presented as follows,

22 $${G}_{c}=3.2945+\frac{0.6450}{{s}^{0.7624}}+3.8051{s}^{0.1023}$$

The resulting transfer function of the closed-loop process is found to be

23 $${G}_{CL}\left(s\right)= \frac{{5.2815s}^{0.8647}+4.5728{s}^{0.7624}+0.89526}{57.19{s}^{1.7624}+5.2815{s}^{0.8647}+5.5728{s}^{0.7624}+0.89526}{e}^{-20.9s}$$

### Simulation results

**Fig. 8:**
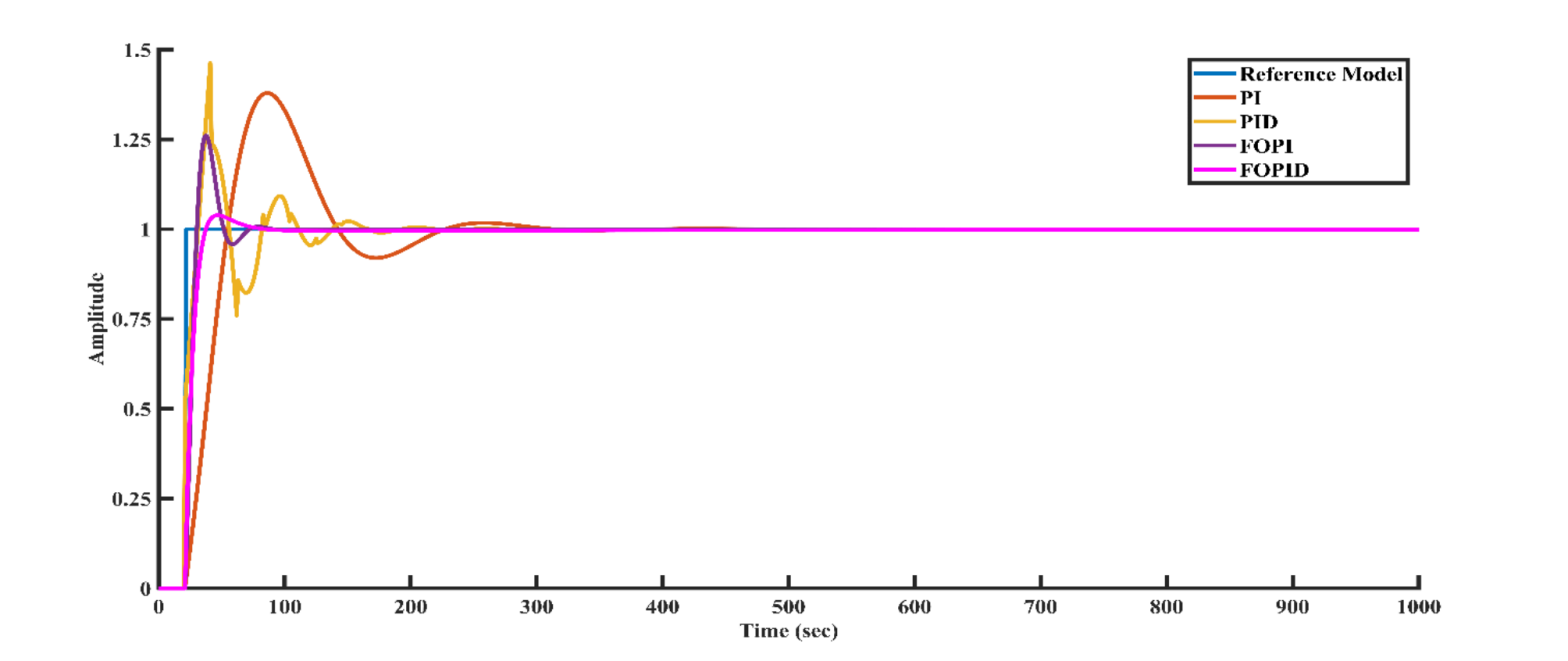
Step response of the reference model and the closed-loop system utilizing all controllers.

Figure 8 illustrates the step response of both the reference model and the closed-loop system using different controllers applied to the SSTLLS. The time-domain analysis and performance metrics for all controllers are detailed in Table 3. Based on the data in Table 3 and Fig. 8, the FOPID controller demonstrates superior performance with a lower rise time of 7.6177 s, a peak time of 17 s, a faster settling time of 41.1456 s, and a lower peak overshoot of 4.0177%. Additionally, it achieves lower ISE and IAE values of 2.3693 and 6.0490, respectively. In contrast, the PID controller shows a rise time of 15.9141 s, a peak time of 41 s, a settling time of 153.665 s, a peak overshoot of 46.2104%, and ISE and IAE values of 8.6362 and 13.5453. Moreover, the AGTM method with the FOPID controller produces the best performance metrics, surpassing other controllers in key areas, which is crucial for real-world applications. The proposed method has also been applied to assess the stability of the SSTLLS system with various controllers, offering additional validation of the results.

**Table 3:** Time domain and performance indices.

Type of controller	Time domain properties					Performance metrics		Stability
	Rise time (s)	Peak time (s)	Settling time (s)	Peak overshoot %	Steady-state error	ISE	IAE	
PI	26.3567	86	214.21	37.9126	4.0442e-09	17.0354	41.7539	1
PID	15.9141	41	153.665	46.2104	1.0091e-10	8.6362	13.5453	1
FOPI	10.0505	28	65.1723	26.0367	3.1997e-04	4.2338	9.4663	1
FOPID	7.6177	17	41.1456	4.0177	0.0015	2.3693	6.0490	1

**Fig. 9:**
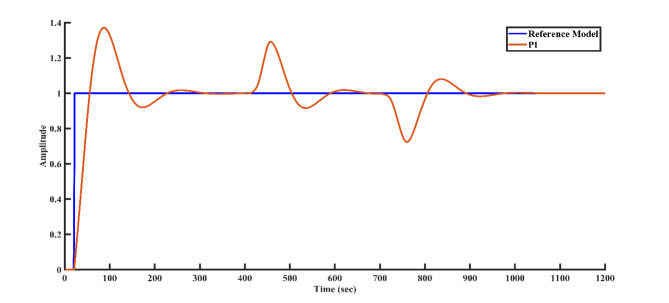
Disturbance rejection of the PI controller and the reference model.

**Fig. 10:**
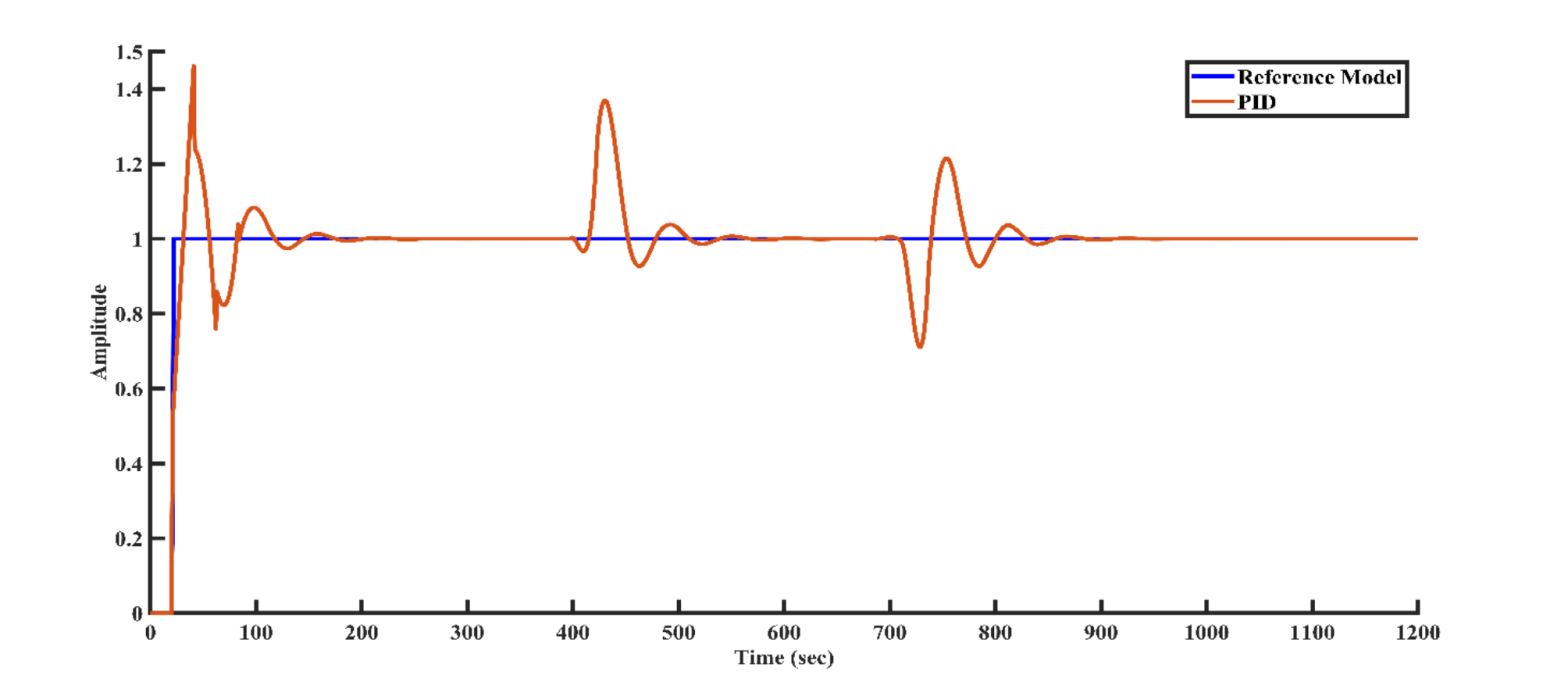
Disturbance rejection of the PID controller and the reference model.

**Fig. 11:**
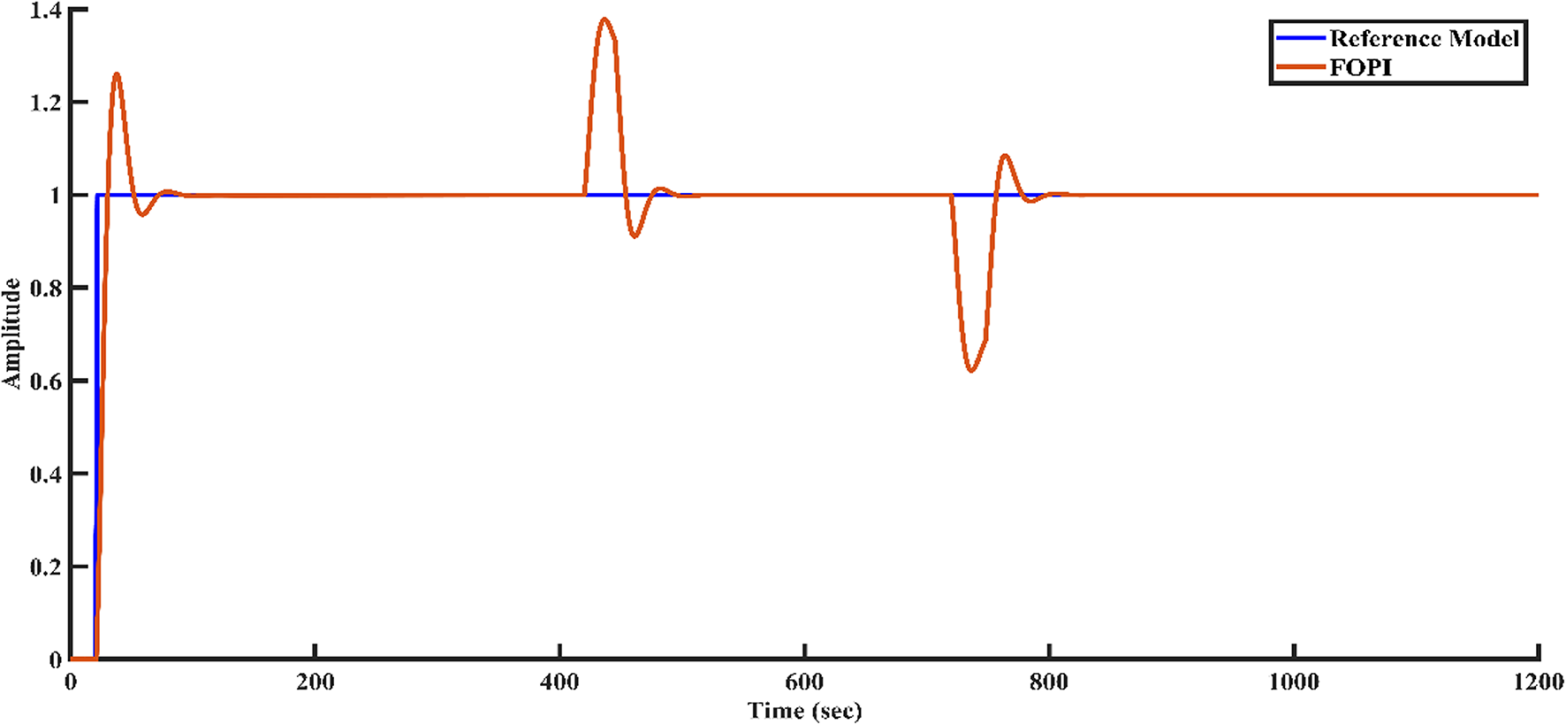
Disturbance rejection of the FOPI controller and the reference model.

**Fig. 12:**
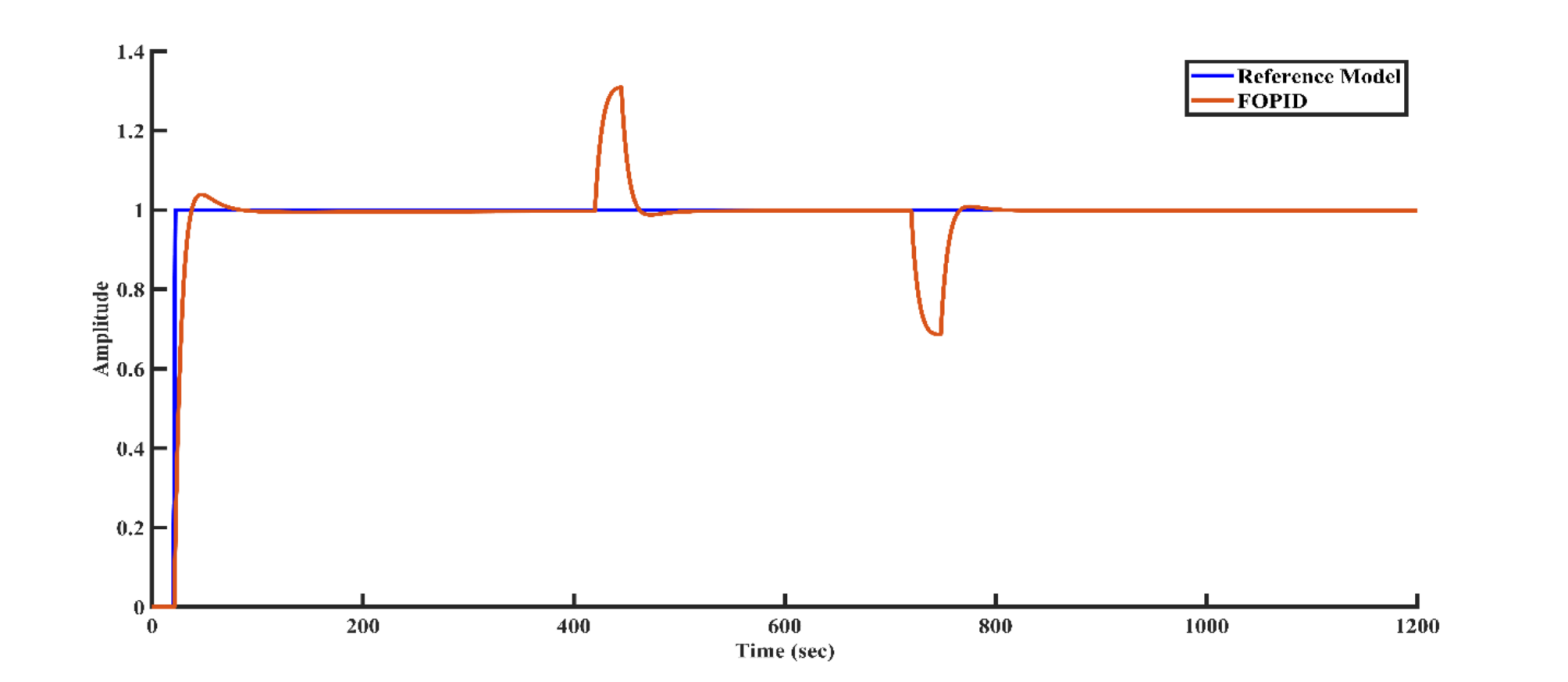
Disturbance rejection of the FOPID controller and the reference model.

To simulate external disturbances that may occur in a real-world plant environment, a disturbance of + 0.3 magnitude is applied to the controller structure at 400 s, followed by a disturbance of -0.3 magnitude at 700 s. Figures 9, 10, 11, and 12 illustrate how each controller structure responds to these disturbances in the step response. Notably, Fig. 12 demonstrates that the FOPID controller provides superior disturbance rejection compared to the other controllers.

**Fig. 13:**
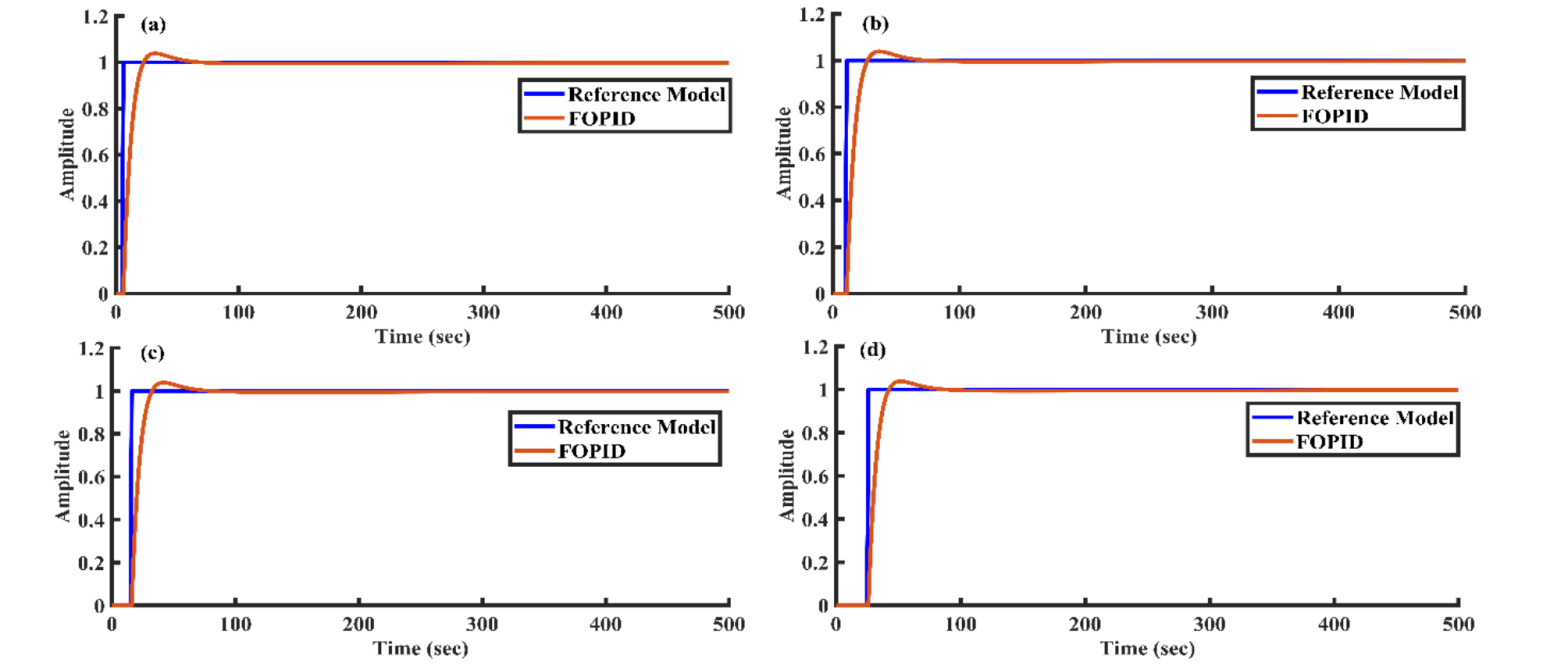
Step response of the FOPID controller and the reference model for different delays: (**a**) 5 s (**b**) 10 s (**c**) 15 s and (**d**) 25 s.

Figure 13 demonstrates the effectiveness of the FOPID controller in handling different delays. The system will likely exhibit a quick rise time, lower settling time, and minimal overshoot across various delays, resulting in improved performance. The response should closely follow the reference model with strong stability.

**Fig. 14:**
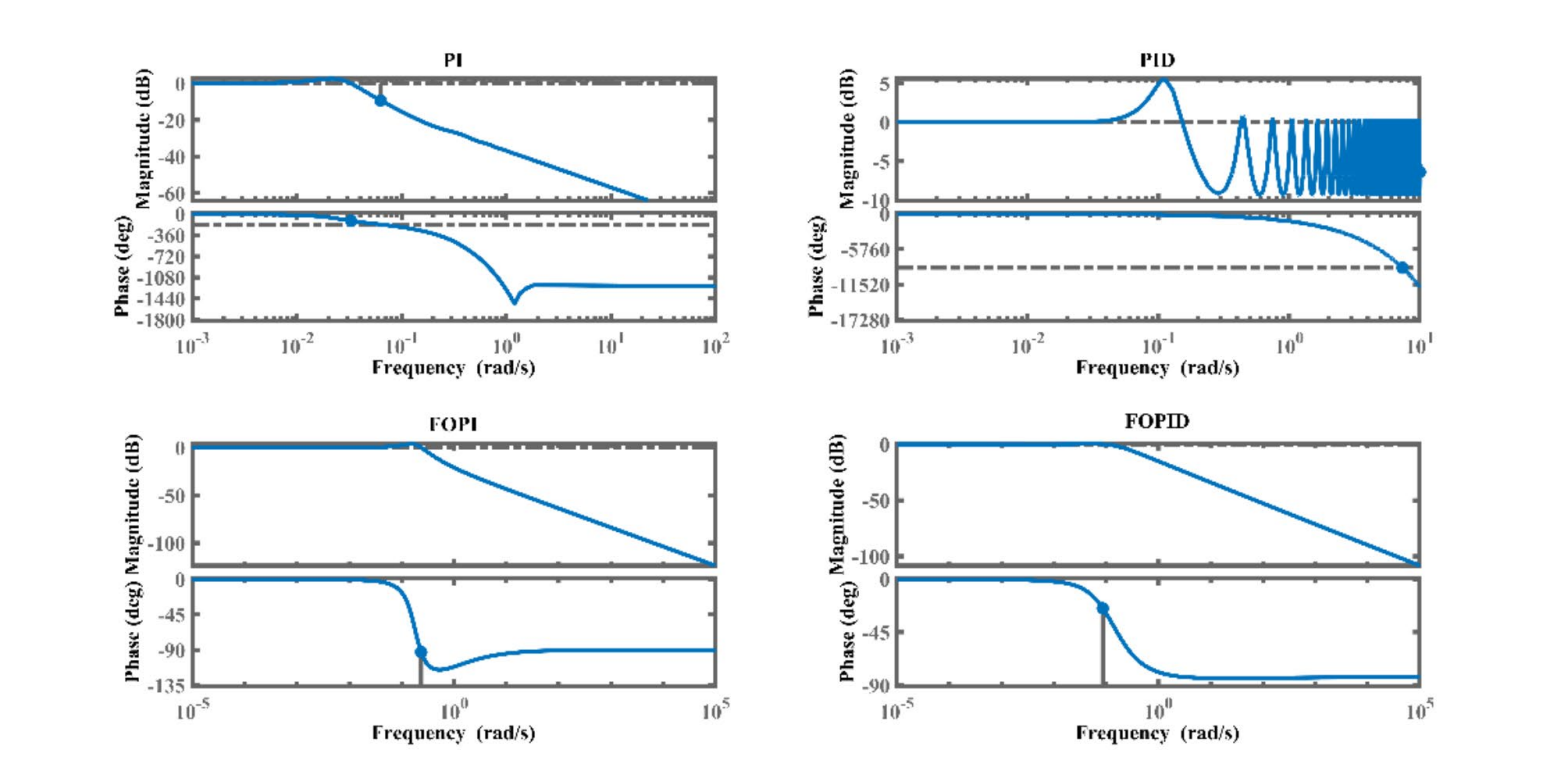
Frequency response of PI, PID, FOPI and FOPID controllers for the SSTLLS.

Additionally, it’s important to consider the frequency domain response specifications associated with PI, PID, FOPI, and FOPID controllers. As demonstrated below, Fig. 14 showcases the frequency domain responses of SSTLLS with different controller configurations. Figure 14 illustrates that the gain margin and phase margin for the FOPID controller increase compared to the other controllers. Table 4 presents the gain margin, phase margin, gain crossover frequency, and phase crossover for PI, PID, FOPI, and FOPID controllers with SSTLLS. It’s clear that increased margins correspond to enhanced system stability.

**Table 4:** Frequency response analysis.

Type of controller	Gain margin (dB)	Phase margin (degrees)	Gain cross-over frequency (w_g_) in rad/s	Phase cross-over frequency (w_*p*_) in rad/s
PI	2.7020	22.0681	0.0668	0.0565
PID	-0.1463	18.2657	4.8501e + 13	7.3570
FOPI	Inf	87.2306	NaN	0.2358
FOPID	Inf	155.2866	NaN	0.0862

### Experimental results

The controllers employing the proposed AGTM optimization method for the SSTLLS are now implemented in real-time across various setpoints to assess their efficacy in managing liquid levels within the system. The controllers’ performance, including tracking accuracy, response time, overshoot, and settling time, was measured and compared across different setpoints and disturbances. Additionally, the implementation of a new Fractional-Order control law in MATLAB/SIMULINK was facilitated for practical purposes. This involved utilizing the FOMCON (Fractional-Order Modeling and Control) toolbox within MATLAB/SIMULINK to implement Fractional-Order controllers, specifying the Fractional-Order of the integral (λ) and derivative (μ)^44^. The Simulink library for Fractional-Order operators provided a structured graphical interface to realize the control algorithms effectively. For the operation of all controllers, the analysis involves varying the setpoints selected at 8, 16, 24, 32, 40, and 45 cm. Regarding changes in load, an initial setpoint of 8 cm was applied to assess the tank’s response within its nonlinear range, and the corresponding readings were recorded. The same approach was replicated for setpoints of 16, 24, 32, and 40 cm, respectively. At each level, a sudden disturbance of five liters of water was introduced by pouring it into the tank until the system regained stability. Water leakage from the tank was considered an error introduced into the system.

### Result of PI controller

#### Variation of the setpoints

**Fig. 15:**
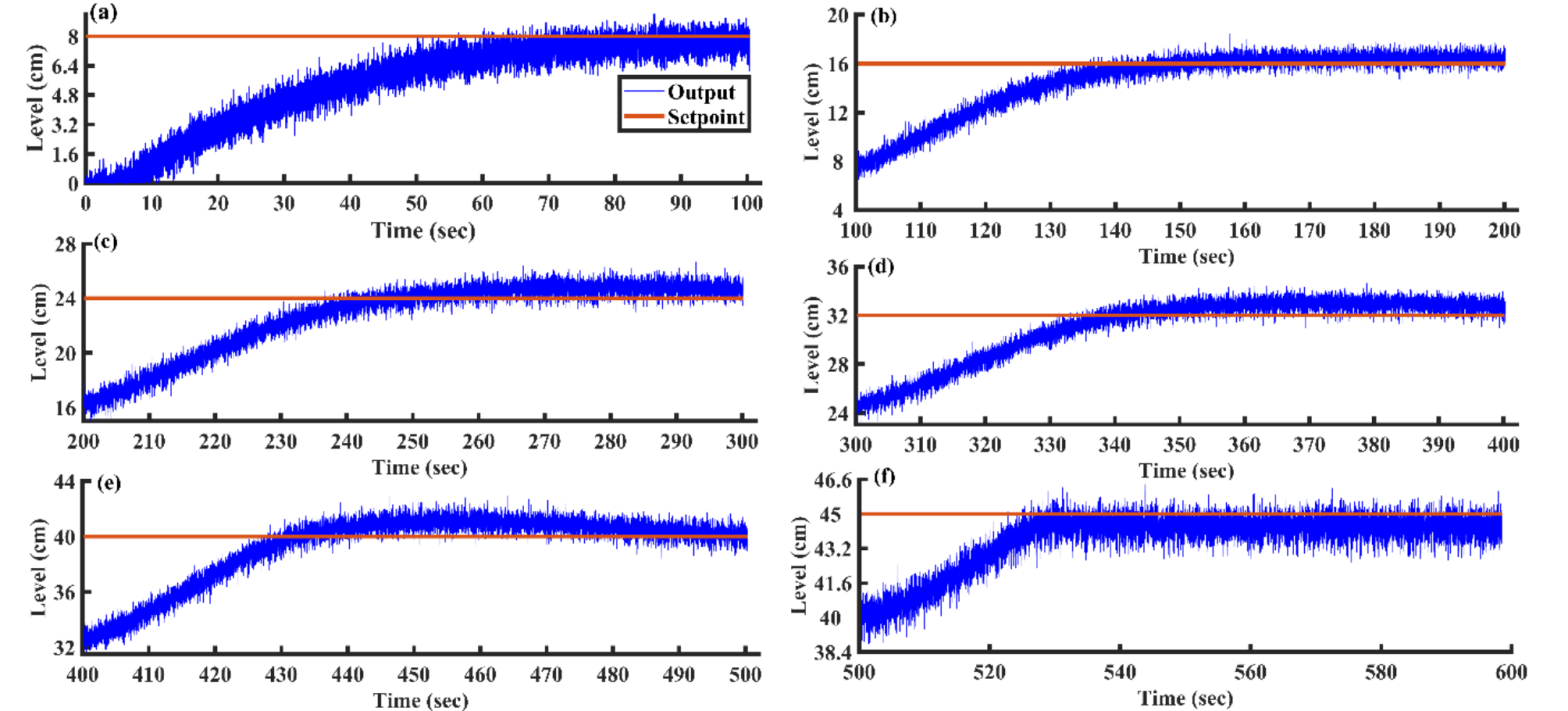
PI Controller servo response for a setpoint change (**a**) 8 cm (**b**) 16 cm (**c**) 24 cm (**d**) 32 cm (**e**) 40 cm and (**f**) 45 cm.

Utilizing the proposed AGTM optimization method, the PI controller is applied to all SSTLLS setpoints. Time-domain and integral performance metrics are evaluated for each setpoint. Figure 15a–f illustrates the servo response characteristics for various setpoints. Across all setpoint intervals, the PI controller consistently exhibits suboptimal performance, as indicated by both time-domain metrics and performance indices. Specifically, the controller’s response times are slower, with longer settling times observed during the setpoint changes. Additionally, the rise time and peak time metrics suggest delayed adjustments to the desired setpoints. In terms of performance indices, such as IAE and ISE, the controller consistently shows higher error values, signaling inefficiency in minimizing deviations from the desired output. This reduction in overall performance highlights the limitations of the PI controller in effectively responding to changes in setpoint demands using the AGTM optimization method.

#### Changes in load

**Fig. 16:**
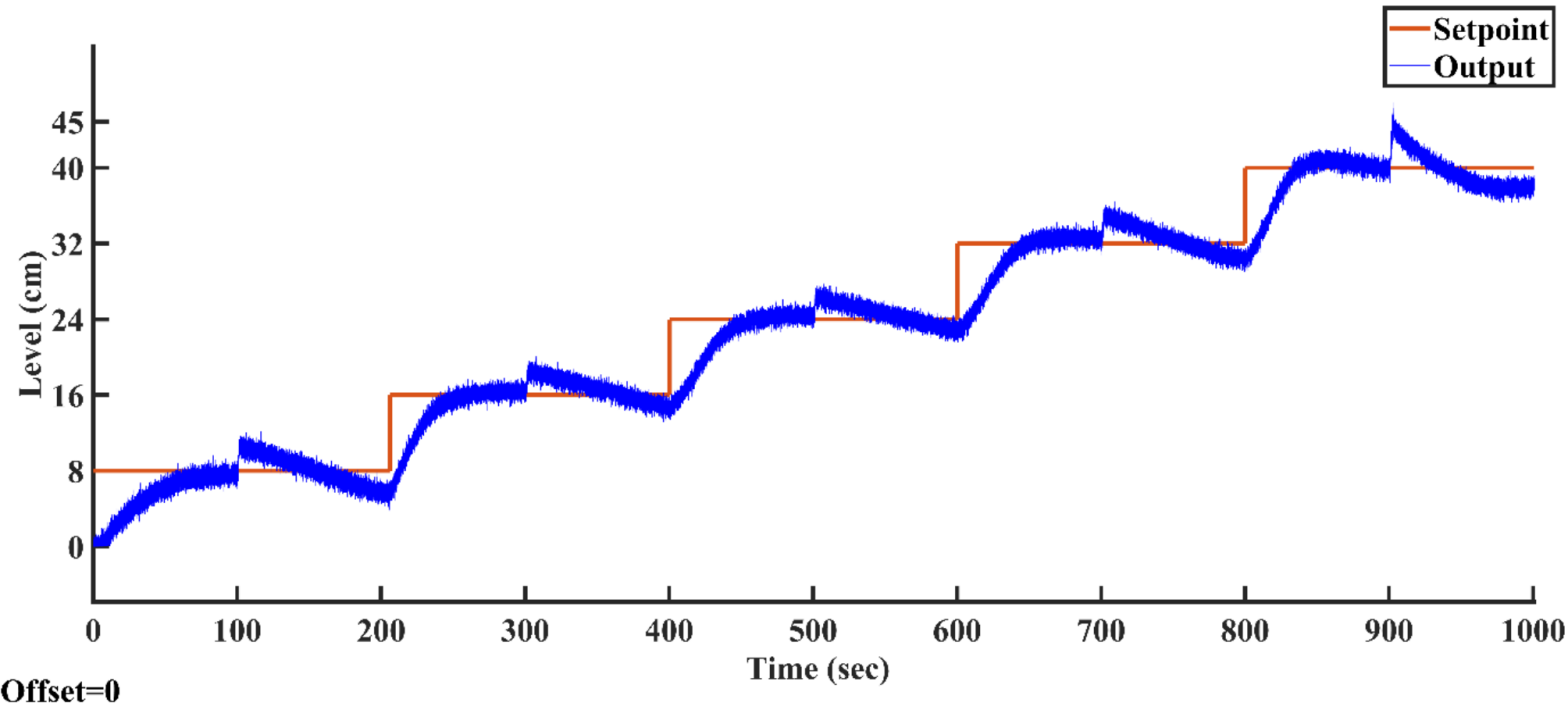
PI Controller regulatory response to changes in load.

The proposed PI controller, based on the AGTM optimization method has been utilized to regulate the SSTLLS level while encountering an external disturbance. Figure 16 showcases the regulatory behavior of the PI controller when subjected to external disturbances for different setpoints. The experimentally modeled SSTLLS is noted for its significant peak overshoot and fluctuating performance in all nonlinear regions, along with prolonged steady-state behavior. It is also evident that the ISE and IAE values are consistently higher across all regions.

### Result of PID controller

#### Variation of the setpoints

**Fig. 17:**
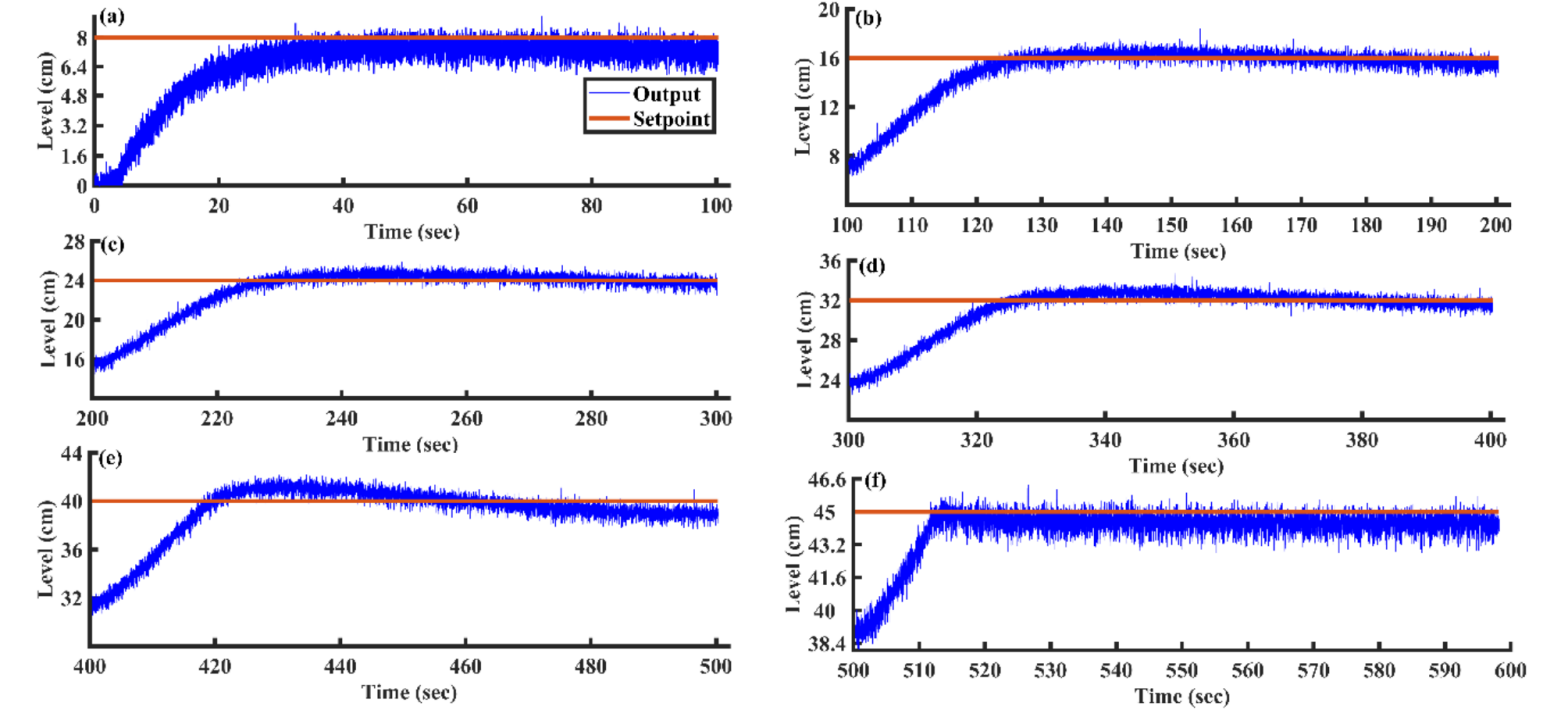
PID Controller servo response for a setpoint change (**a**) 8 cm (**b**) 16 cm (**c**) 24 cm (**d**) 32 cm (**e**) 40 cm and (**f**) 45 cm.

The PID controller operates for the subsequent setpoints, acquiring integral and time-domain performance metrics. The characteristics of the servo response for different setpoint changes are presented in Fig. 17a–f. Throughout all operational regions, the PID controller consistently demonstrates rapid oscillations, resulting in greater maximum deviations from the setpoint, longer response times, and extended oscillation periods. Additionally, the ISE and IAE values remain elevated across these regions.

#### Changes in load

**Fig. 18:**
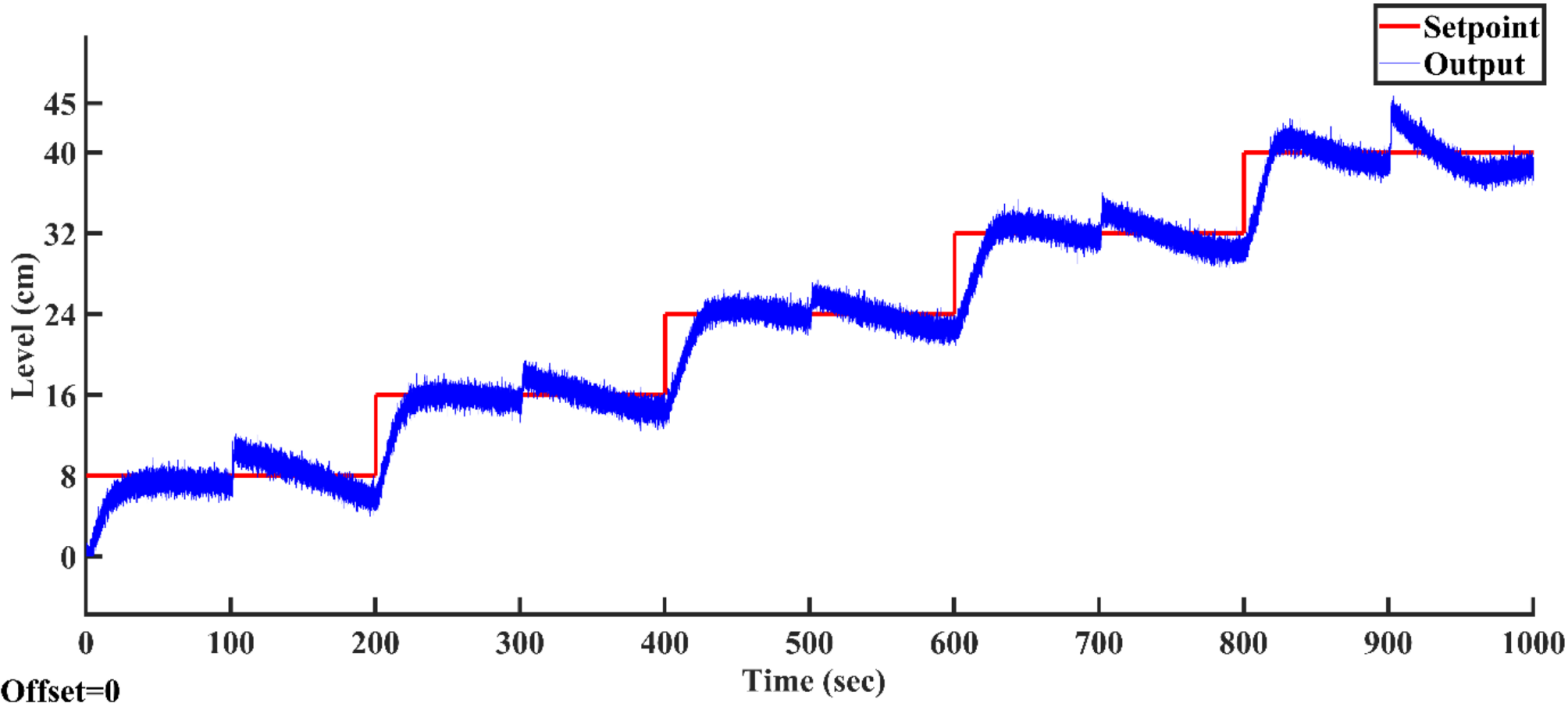
PID Controller regulatory response to changes in load.

The AGTM-optimized PID controller has been used to regulate the SSTLLS level amid external disturbances. Figure 18 shows the controller’s effectiveness in managing disturbances at different setpoints, with ISE and IAE values remaining consistently high across all ranges.

### Result of FOPI controller

#### Variation of the setpoints

**Fig. 19:**
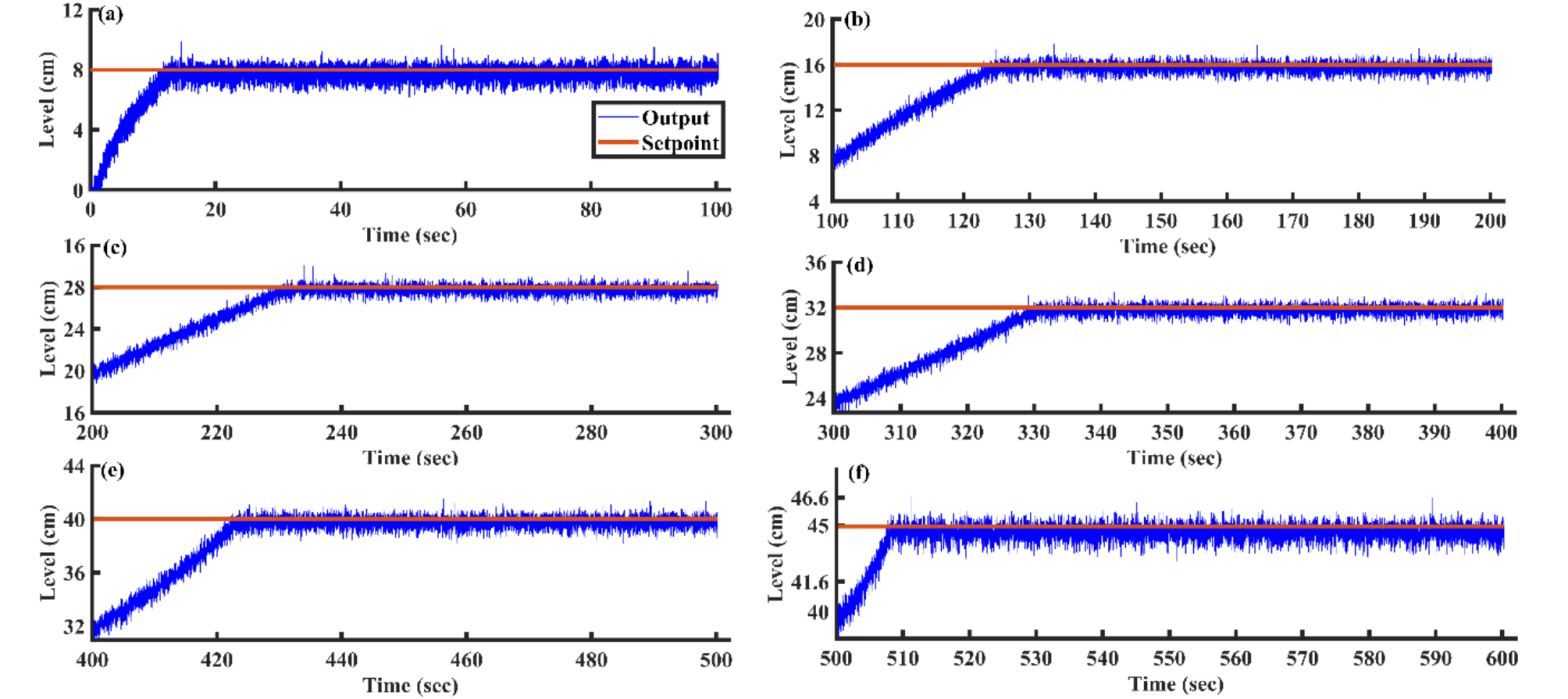
FOPI Controller servo response for a setpoint change (**a**) 8 cm (**b**) 16 cm (**c**) 24 cm (**d**) 32 cm (**e**) 40 cm and (**f**) 45 cm.

The FOPI controller was evaluated at all setpoints, documenting both time-domain specifications and performance indices. Figure 19a–f illustrates the servo response for various setpoint profiles. Across all ranges, the FOPI controller shows superior performance, reflected by lower time-domain values and improved performance indices.

#### Changes in load

**Fig. 20:**
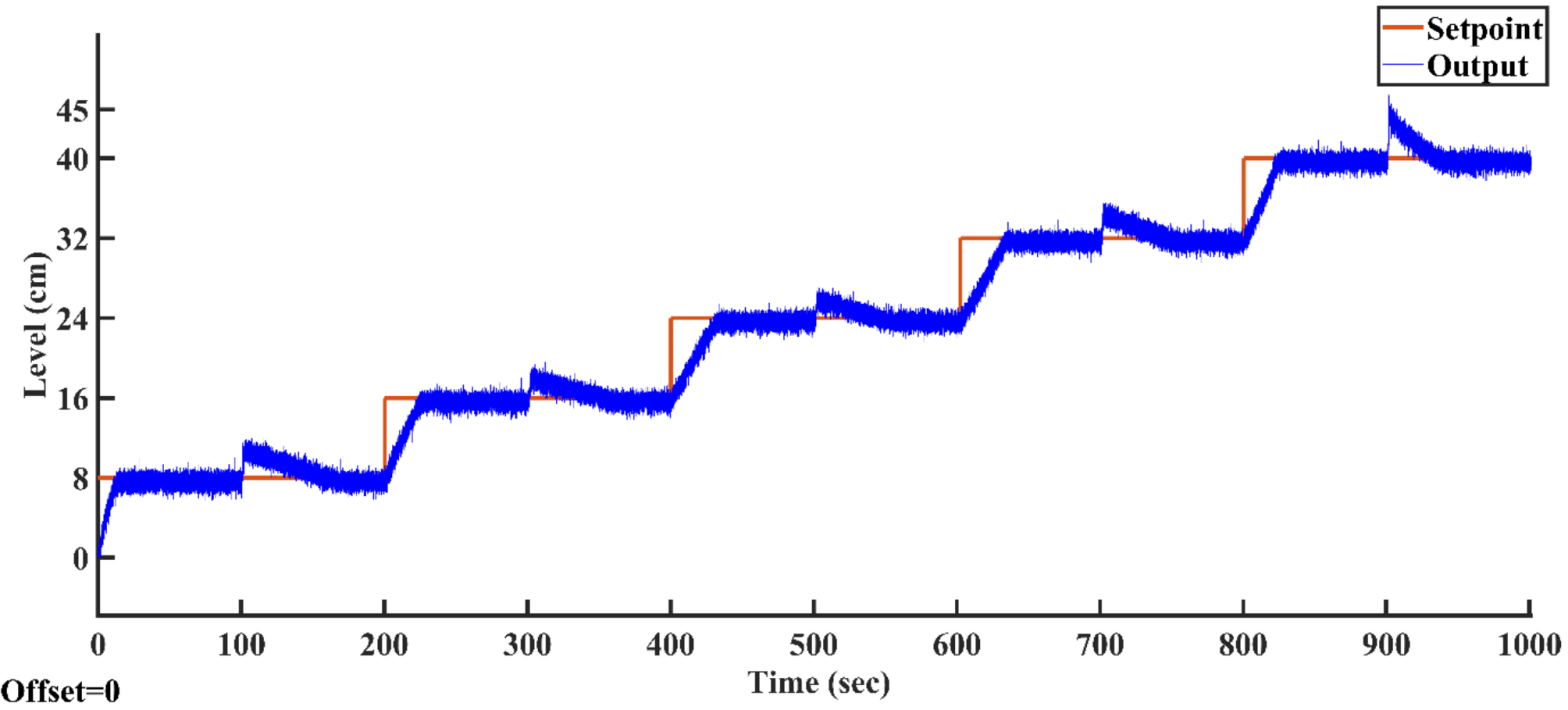
FOPI Controller regulatory response to changes in load.

The AGTM optimization-based FOPI controller regulates the SSTLLS level amid external disturbances. Figure 20 shows the controller’s response to these disturbances over various intervals, with performance metrics recorded for each setpoint. The FOPI controller consistently demonstrates superior performance, as shown by lower time-domain values and improved performance indices.

### Result of FOPID controller

#### Variation of the setpoints

**Fig. 21:**
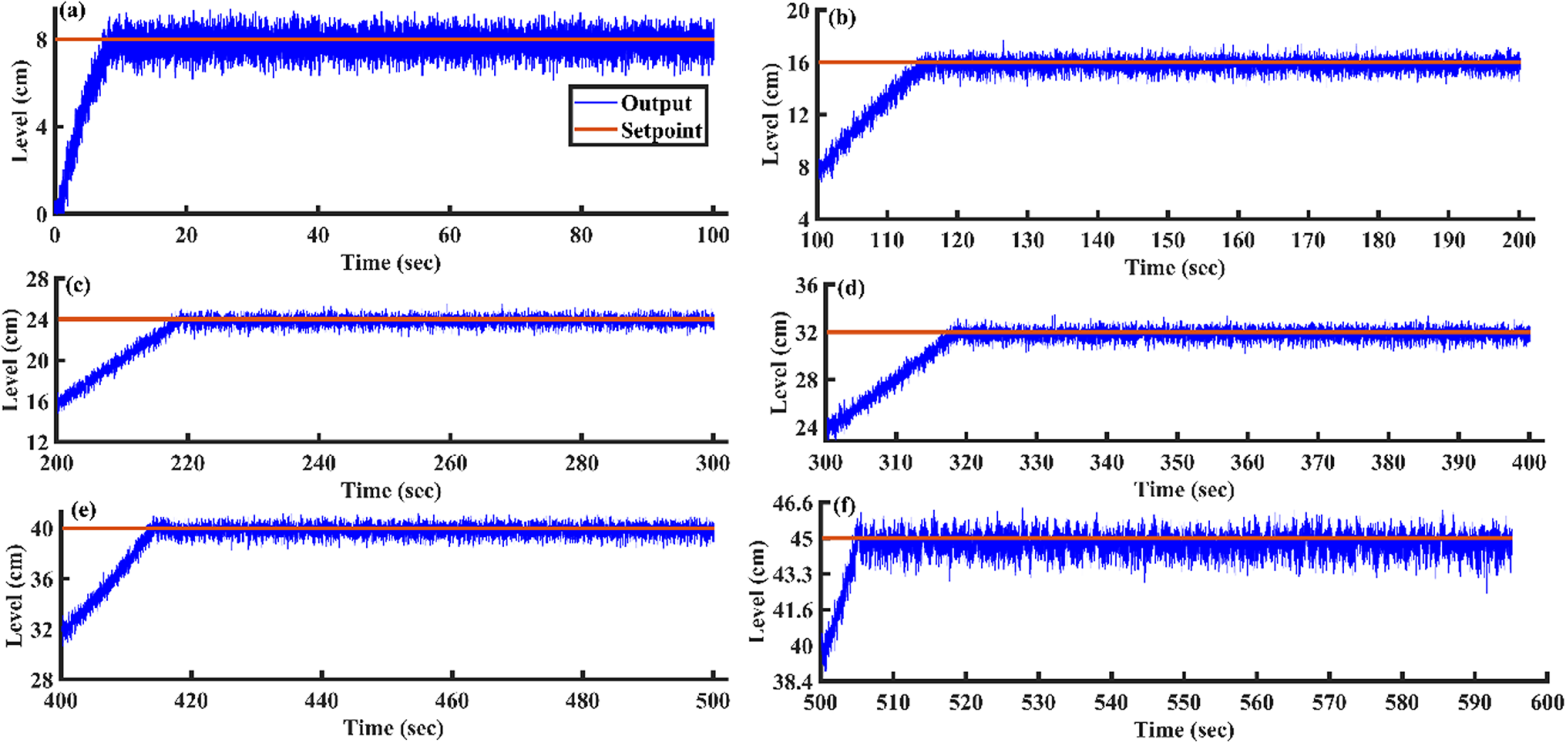
FOPID Controller servo response for a setpoint change (**a**) 8 cm (**b**) 16 cm (**c**) 24 cm (**d**) 32 cm (**e**) 40 cm and (**f**) 45 cm.

After optimizing the controller values using the AGTM method, the FOPID controller operates at the subsequent setpoints, capturing integral and time-domain performance. Figure 21a–f shows the servo response characteristics for different setpoint change profiles. The FOPID controller consistently demonstrates positive results in time-domain analysis and performance metrics across all specified setpoint ranges.

#### Changes in load

**Fig. 22:**
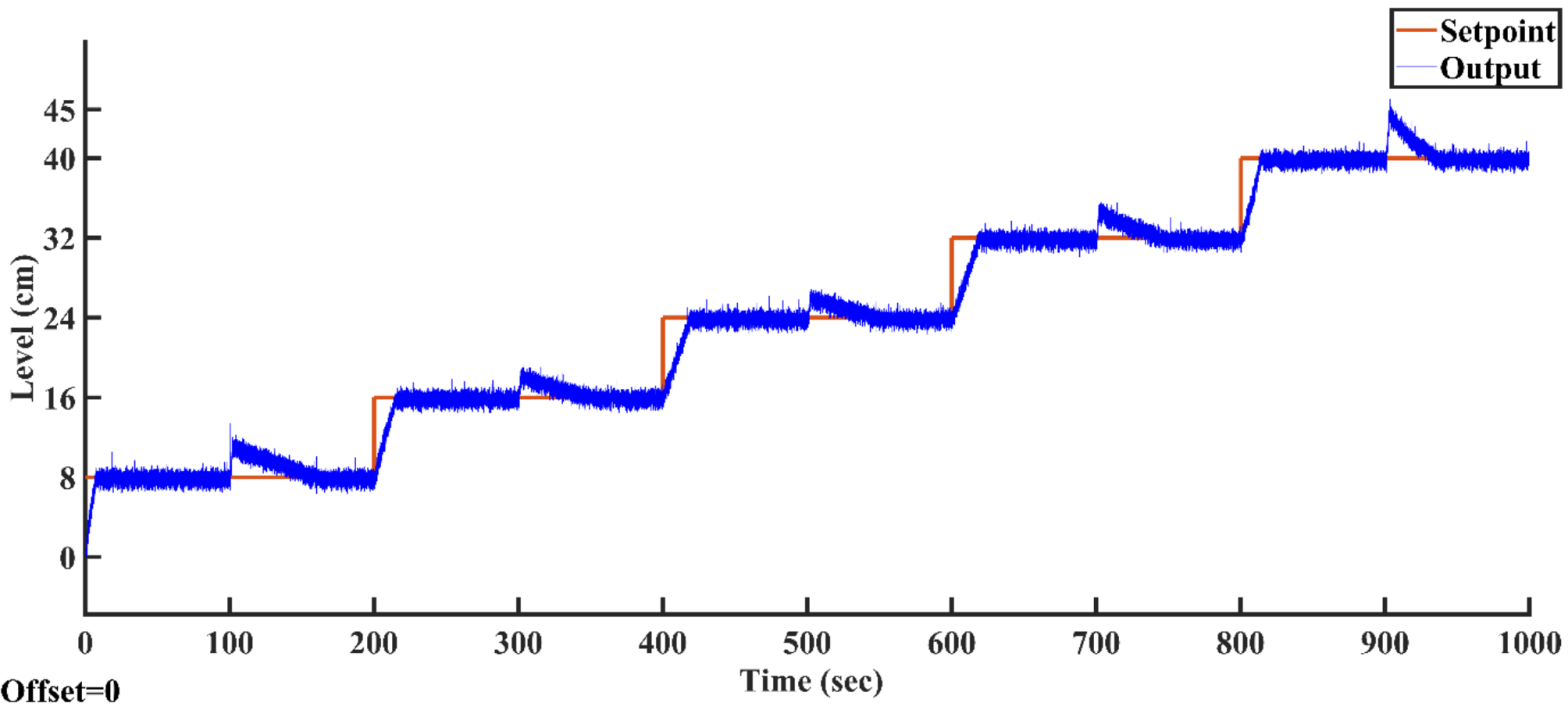
FOPID Controller regulatory response to changes in load.

The proposed AGTM optimization method-based FOPID controller has been utilized to regulate the SSTLLS level while encountering external disturbances. Figure 22 illustrates how the FOPID controller responds to external disturbances across different intervals. Both ISE and IAE values consistently demonstrate lower levels throughout all the designated intervals.

### Result analysis of all controllers

**Fig. 23:**
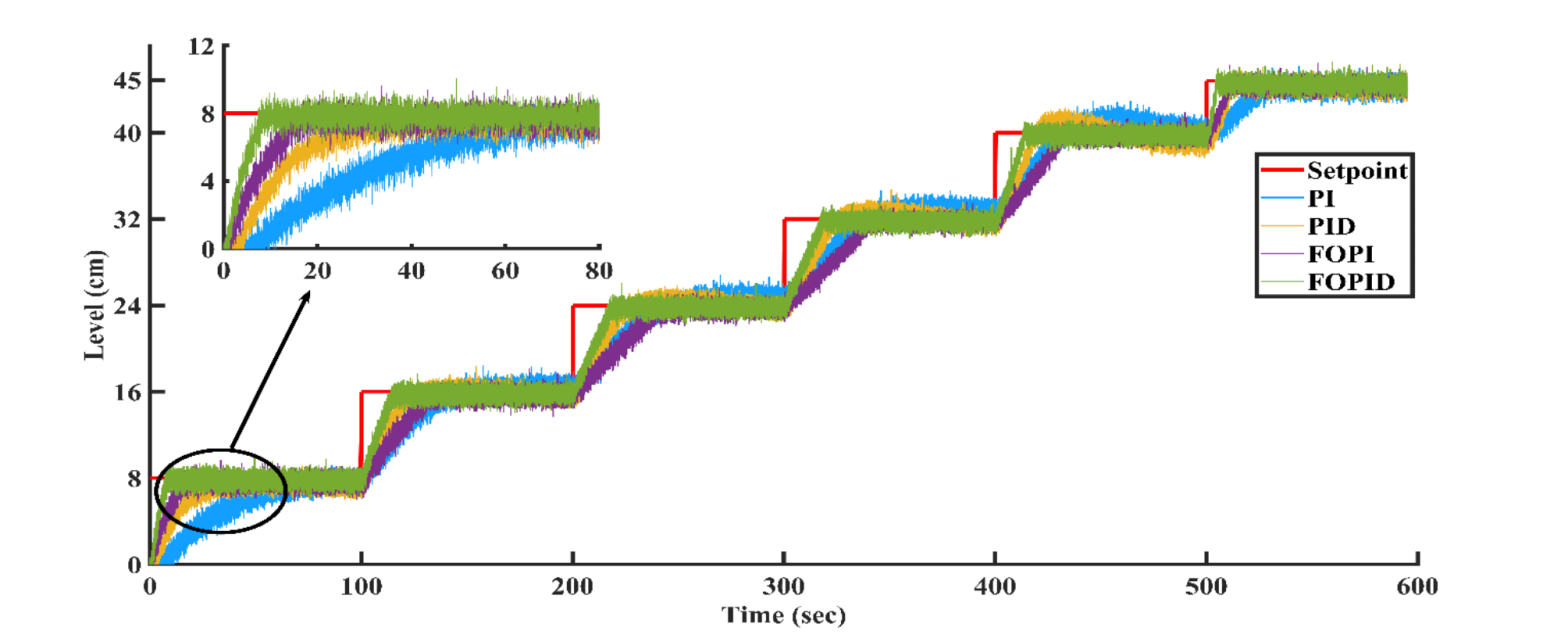
All Controller’s servo response across various setpoint changes.

Figure 23 illustrates the output trajectories of the servo response of all controllers across different setpoints. In this figure, the FOPID controller, optimized using the AGTM technique, is compared with the FOPI, PID, and PI controllers across different setpoint changes. The FOPID controller exhibits notably faster tracking of the setpoint compared to the other controllers. Its performance surpasses that of the traditional PI, PID, and FOPI controllers in regulating the liquid level in the SSTLLS. Table 5 details the time-domain analysis and performance metrics for all controllers across these diverse setpoints. The table clearly shows that the rise time, peak time, settling time, peak overshoot, and steady-state error for various setpoints are notably decreased when employing the FOPID controller compared to other controllers. This improvement is particularly noticeable in regions characterized by a higher level of nonlinearity. This indicates that the FOPID controller effectively maintains control over the liquid level with superior efficiency and accuracy.

**Table 5:** Comparison of time domain analysis and performance indices of all controllers in servo response across different setpoints.

Setpoint	Type of controller	Time domain specifications					Performance indices	
		Rise time (s)	Peak time (s)	Settling time (s)	Peak overshoot (%)	Steady-state error	ISE	IAE
(0–8) cm	PI	58.0968	85.952	88.4	39.7413	0.0497	20.4156	32.9943
	PID	32.932	71.98	82.82	27.3732	0.1016	8.9673	18.2147
	FOPI	11.988	14.464	31.036	24.5635	0.0095	3.8642	8.9626
	FOPID	7.348	12.744	28.06	20.4758	0.0269	2.8830	7.0800
(8–16) cm	PI	37.204	57.612	69.748	26.1253	0.0700	12.8533	22.1811
	PID	28.812	54.388	65.188	18.3030	0.0563	8.7846	14.8441
	FOPI	23.7	33.692	39.516	14.3494	0.0524	8.6017	15.1100
	FOPID	14.586	26.52	29.416	12.0933	0.0235	5.3262	10.3871
(16–24) cm	PI	38.66	69.896	76.296	12.5597	0.1477	13.1780	24.3348
	PID	24.808	49.624	57.952	9.3799	0.0381	10.0363	19.1727
	FOPI	21.888	34	40.716	7.5917	0.0316	11.1521	17.5318
	FOPID	17.872	23.284	28.98	5.5612	0.0005885	6.6258	12.1051
(24–32) cm	PI	35.916	78.876	83.212	12.5362	0.0269	12.0891	24.1502
	PID	29.04	50.804	58.624	9.9296	0.5130	9.9811	19.7235
	FOPI	24.924	42	46.804	7.5845	0.0053	8.6462	17.8386
	FOPID	17.628	32.548	39.388	4.6826	0.1451	6.8855	12.3215
(32–40) cm	PI	28.676	64.076	92.92	9.0216	0.0616	10.4672	21.5394
	PID	25.956	33.228	89.836	8.3129	0.1153	9.3442	18.3549
	FOPI	22.156	26.332	59.308	4.9679	0.0561	8.4648	15.7996
	FOPID	13.316	21.132	32.476	3.4150	0.0127	5.6649	10.4937
(40–45) cm	PI	27.66	43.768	75.592	8.4949	0.0212	4.7590	14.7288
	PID	11.948	26.652	58.424	5.1294	0.1195	3.3346	10.7107
	FOPI	8.012	11.32	34.566	3.8214	0.0011	1.7052	7.0963
	FOPID	4.9	7.804	27.456	3.0240	0.0005108	1.1101	5.4654

According to Table 5 and Fig. 23, in the highly nonlinear lower region with a setpoint of 0–8 cm, the FOPID controller achieves a lower rise time of 7.348 s, a lower peak time of 12.744 s, a faster settling time of 28.06 s, a lower peak overshoot of 20.4758%, and lower ISE and IAE values of 3.8642 and 89,626, respectively. In comparison, the PID controller with the same setpoint has a rise time of 32.932 s, a peak time of 71.98 s, a settling time of 82.82 s, a peak overshoot of 27.3732%, and ISE and IAE values of 8.9673 and 18.2147, respectively. Similarly, in the highly nonlinear upper region with a setpoint of 40–45 cm, the FOPID controller shows a lower rise time of 4.9 s, a lower peak time of 7.804 s, a faster settling time of 27.456 s, a lower peak overshoot of 3.024%, and lower ISE and IAE values of 1.1101 and 5.4654, respectively, compared to the PID controller, which has a rise time of 11.948 s, a peak time of 26.652 s, a settling time of 58.424 s, a peak overshoot of 5.1294%, and ISE and IAE values of 3.3346 and 10.7107, respectively. The highly nonlinear regions, specifically between 0–8 cm and 40–45 cm, show exceptionally low ISE and IAE values, which further emphasize the superior efficiency of the FOPID controller compared to other controllers. This high performance is consistently evident across all other setpoints in both time-domain responses and performance metrics.

Additionally, we compared our results with those found in the literature. Table 6 presents a comprehensive comparison of the ISE and IAE values obtained from both servo and regulatory responses at different setpoints in the current study, as well as those from a previous study cited as^45^. It is important to highlight that the referenced study used a PI controller, which was tuned using the Skogestad Internal Model Control (SIMC) tuning method. A thorough analysis of Table 6 reveals that in the highly nonlinear lower region, with a setpoint of 0–8 cm, the FOPID controller achieves lower ISE and IAE values for the servo response, which are 2.8830 and 7.0800, respectively. For the regulatory response, the ISE and IAE values are 5.6208 and 19.2696, respectively. In contrast, the SIMC-PI controller from reference^45^ shows significantly higher ISE and IAE values for the servo response at the same setpoint, 186.4216 and 244.9153, respectively, and the regulatory response, 1364.222 and 6145.408, respectively. Similarly, in the highly nonlinear upper region, with a setpoint of 32–40 cm, the FOPID controller again demonstrates lower ISE and IAE values in the servo response, with values of 1.1101 and 5.4654, respectively. For the regulatory response, the values are 37.94 and 99.0582, respectively. In comparison, the SIMC-PI controller^45^ shows much higher ISE and IAE values at the same setpoint, with 186.6177 and 227.1456 for the servo response, and 13,238.23 and 4645.25 for the regulatory response. This pattern is consistently observed across all other setpoints in the performance indices. It is clear that the ISE and IAE values for both servo and regulatory responses are significantly lower in the current study than those reported in reference^45^. This contrast indicates that the approach used in the current research offers superior performance in reducing error metrics, highlighting its effectiveness over the previously used PI controller with SIMC optimization.

**Table 6:** Comparison of performance indices in servo and regulatory response.

Setpoint	Type of controller	Performance indices in servo response		Performance indices in regulatory response	
		ISE	IAE	ISE	IAE
(0–8) cm	SIMC-PI^45^	186.4216	244.9153	1364.222	6145.408
	FOPID	2.8830	7.0800	5.6208	19.2696
(8–16) cm	SIMC-PI^45^	116.6536	196.1724	3388.292	2654.566
	FOPID	5.262	10.3871	12.1502	37.3256
(16–24) cm	SIMC-PI^45^	154.9183	226.5198	3388.292	2654.566
	FOPID	6.6258	12.1051	20.0262	78.0615
(24–32) cm	SIMC-PI^45^	106.7335	187.0507	13238.23	4645.26
	FOPID	5.6649	10.4937	28.674	78.0615
(32–40) cm	SIMC-PI^45^	186.6177	227.1456	13238.23	4645.25
	FOPID	1.1101	5.4654	37.94	99.0582

## Conclusion

The current research in this work proposes an optimization approach for a nonlinear real-time process (SSTLLS in particular) employing AGTM optimization technique to achieve a closed-loop structure response that closely resembles a reference model. The novelty of this work lies in the recommendation of the AGTM optimization algorithm for tuning the Fractional-Order controller parameters and evaluating the performance of the AGTM optimization technique on the nonlinear process. To address stabilization challenges, an accurate reference model, defined by a transfer function based on time-domain parameters, was extensively used. This model incorporates plant dynamics, enabling the easy generation of controllers of any order and offering flexibility in selecting expansion points for optimal response matching, with no restrictions on controller or model orders. In both simulations and real-time experiments, Fractional Order and Integer-Order controllers were developed and applied to regulate the SSTLLS for various setpoints. These comparisons, conducted for both setpoint and load changes, reveal distinct patterns corresponding to different regions of nonlinearity. The Fractional-Orders (λ and µ) provide extra flexibility for fine-tuning the system’s response, making Fractional-Order controllers more adaptable to nonlinear dynamics, like those in SSTLLS. This flexibility improves setpoint tracking, disturbance rejection, and stability. Unlike Integer-Order controllers, which may require retuning, Fractional-Order controllers can better handle parameter changes and disturbances, offering robust performance. Experimental results show that the AGTM-based Fractional-Order controllers effectively reject load disturbances and maintain stability across varying system parameters.

## Data Availability

The datasets used and/or analyzed during the current study are available from the corresponding author upon reasonable request.
